# Exosomes derived from human adipose mesenchymal stem cells ameliorate hepatic fibrosis by inhibiting PI3K/Akt/mTOR pathway and remodeling choline metabolism

**DOI:** 10.1186/s12951-023-01788-4

**Published:** 2023-01-25

**Authors:** Zilong Zhang, Jin Shang, Qinyan Yang, Zonglin Dai, Yuxin Liang, Chunyou Lai, Tianhang Feng, Deyuan Zhong, Haibo Zou, Lelin Sun, Yuhao Su, Su Yan, Jie Chen, Yutong Yao, Ying Shi, Xiaolun Huang

**Affiliations:** 1 Liver Transplantation Center and HBP Surgery, Sichuan Cancer Hospital & Institute, Sichuan Cancer Center, Cancer Hospital Affiliate to University of Electronic Science and Technology of China, Chengdu, 610042 Sichuan China; 2 School of Medicine, University of Electronic Science and Technology of China, Chengdu, 610054 Sichuan China; 3 Department of Core laboratory, Sichuan Provincial People’s Hospital, University of Electronic Science and Technology of China, Chengdu, 610072 Sichuan China

**Keywords:** Liver fibrosis, Human adipose mesenchymal stem cells, Exosomes, Metabolomics, Choline

## Abstract

**Abstract:**

Liver fibrosis is a chronic liver disease with the presence of progressive wound healing response caused by liver injury. Currently, there are no approved therapies for liver fibrosis. Exosomes derived from human adipose mesenchymal stem cells (hADMSCs-Exo) have displayed a prominent therapeutic effect on liver diseases. However, few studies have evaluated therapeutic effect of hADMSCs-Exo in liver fibrosis and cirrhosis, and its precise mechanisms of action remain unclear. Herein, we investigated anti-fibrotic efficacy of hADMSCs-Exo in vitro and in vivo, and identified important metabolic changes and the detailed mechanism through transcriptomic and metabolomic profiling. We found hADMSCs-Exo could inhibit the proliferation of activated hepatic stellate cells through aggravating apoptosis and arresting G1 phase, effectively inhibiting the expression of profibrogenic proteins and epithelial-to-mesenchymal transition (EMT) in vitro. Moreover, it could significantly block collagen deposition and EMT process, improve liver function and reduce liver inflammation in liver cirrhosis mice model. The omics analysis revealed that the key mechanism of hADMSCs-Exo anti-hepatic fibrosis was the inhibition of PI3K/AKT/mTOR signaling pathway and affecting the changes of metabolites in lipid metabolism, and mainly regulating choline metabolism. CHPT1 activated by hADMSCs-Exo facilitated formation and maintenance of vesicular membranes. Thus, our study indicates that hADMSCs-Exo can attenuate hepatic stellate cell activation and suppress the progression of liver fibrosis, which holds the significant potential of hADMSCs-Exo for use as extracellular nanovesicles-based therapeutics in the treatment of liver fibrosis and possibly other intractable chronic liver diseases.

**Graphical Abstract:**

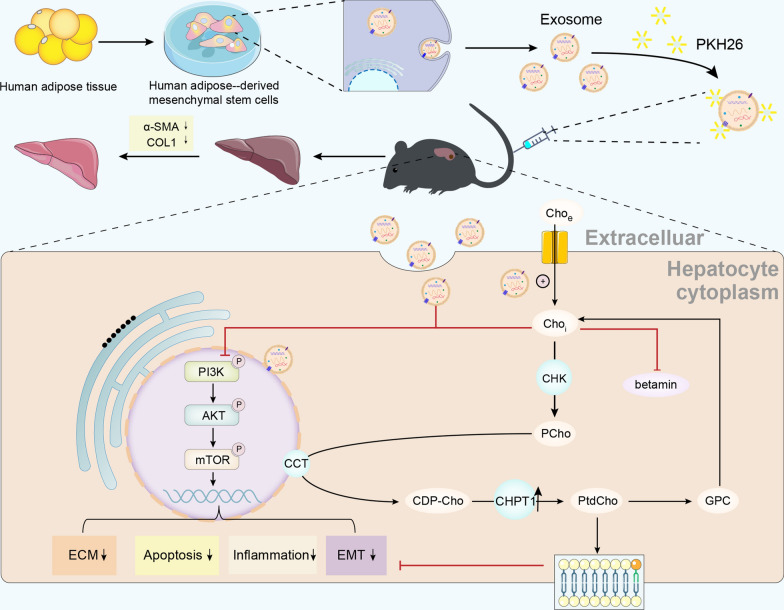

**Supplementary Information:**

The online version contains supplementary material available at 10.1186/s12951-023-01788-4.

## Introduction

Liver fibrosis is one of the major causes of morbidity and mortality worldwide, and the global burden of liver fibrosis is expected to increase in the coming years [1]. It is a chronic liver disease with the presence of persistent inflammatory response as well as progressive wound healing response caused by liver injury [2]. During the progression of liver fibrosis, further aggravation of inflammatory cell recruitment, myofibroblast activation, and excessive extracellular matrix (ECM) deposition may culminate in cirrhosis and liver cancer [3, 4]. The process of liver fibrosis is also closely linked to metabolism. A profound metabolic reprogramming, including a shift towards aerobic glycolysis and excessive accumulation of lipids, was recently identified as an essential mechanism and hallmark in hepatic stellate cell (HSCs) activation, leading to pathological liver fibrosis [5–7]. However, until now, there are no FDA-approved drugs and effective therapies for the treatment of liver cirrhosis except liver transplantation [8]. Owing to liver cirrhosis-associated complications, donor shortage, and the requirement for lifelong immunosuppression, only a small percentage of patients with cirrhosis benefit from liver transplants [9]. Therefore, liver regeneration therapy has emerged as an attractive therapeutic strategy.

Recently, mesenchymal stem cells (MSCs) as well as their derivates provide an alternative for patients with liver disorders. Various stem cells derived from human bone marrow (BMSCs), umbilical cord (UCSCs), and adipose tissue (ADSCs) have been utilized in the treatment of liver disease [10–13]. Both clinical and experimental evidence has shown that MSCs could efficiently protect hepatocytes by transdifferentiating into functional hepatocytes, secreting a wide range of immunosuppressive and trophic factors, and exosomes through autocrine or paracrine effects, leading to an antifibrotic effect and promote liver regeneration [11, 14]. Nevertheless, MSCs transplantations have numerous limitations including substantial cell death, poor engraftment upon transplantation and iatrogenic tumor formation [15]. Interestingly, previous studies revealed that MSCs acted as “conducting cells” to regulate host cells including macrophages and improve the local microenvironment mainly via extracellular vesicle (EV) or exosome signals to exert unique a antifibrotic effect [11, 16–18]. In an attempt to enhance the effects of stem cell therapeutics, our group has suggested MSCs-derived exosomes as potential therapeutic tools for liver diseases [19, 20].

Exosomes are physiological nano-sized membrane vesicles (40–150 nm diameter) with a lipid bilayer membrane of endocytic origin from donor cells. It contains biologically active proteins, lipids and nucleic acids such as miRNAs, mRNAs, and DNA, which can be internalized into recipient cells [21, 22]. Growing evidence suggests that the exosomes derived from BMSCs and UCSCs demonstrate an improved therapeutic effect in treating acute and chronic liver injuries, and exosomes derived from ADSCs have a particular advantage in the treatment of chronic liver disease [23–25]. Importantly, it has been reported that human adipose mesenchymal stem cells-derived EVs successfully inhibited the activity of activated hepatic stellate cells (HSCs) and functionally alleviated liver fibrosis in the thioacetamide-induced liver fibrosis mice model [25]. However, the detailed mechanism remains unclear.

In this study, we utilized the experimental mouse model of liver cirrhosis and activated HSCs to investigate the effects of hADMSCs-Exo on carbon tetrachloride (CCl_4_)-induced liver fibrosis and transforming growth factor-β1 (TGF-β1)-induced activated HSCs, respectively. We confirmed that hADMSCs-Exo could ameliorate HSCs activation and suppres the progression of liver fibrosis. Importantly, we combined transcriptomic and metabolomic studies to identify important metabolic processes and detailed mechanism. Our results demonstrated that hADMSCs-Exo ameliorated hepatic fibrosis mainly by restoring the abnormal choline metabolism and inhibiting PI3K/Akt/mTOR Pathway.

## Materials and methods

### Preparation and culture of hADMSCs

Liposuction aspirates from subcutaneous adipose tissue sites were obtained from 10 young women (aged 20–30 years) who received liposuction from January to May 2020 at the Sichuan Provincial People’s Hospital. The study protocol was approved by Sichuan Academy of Medical Sciences and Sichuan Provincial People’s Hospital ethics committee (NO2021-448). Informed consent was obtained from each subject. hADMSCs were isolated, as described previously [26]. In brief, adipose tissues were washed three to four times with phosphate-buffered saline (PBS) supplemented with penicillin–streptomycin, then the tissue was mechanically chopped before digestion with 0.75 mg/ml collagenase type I (BioFroxx) at 37 °C and 225 rpm orbital shaking inside a MaxQ 8000 shaking incubator for 30 min. The digested tissue was washed with Dulbecco’s modified Eagle’s medium (DMEM) F12 containing 10% fetal bovine serum (FBS) and centrifuged at 300 *g* for 5 min. The cell pellet was resuspended in DMEM F12 supplemented with 10% FBS in a 37 °C incubator with 5% CO2. The hADMSCs from passages 3 to 5 were applied in this study.

Then hADMSCs were identified with fluorescein isothiocyanate (FITC)-conjugated antibodies against CD90, allophycocyanin (APC)-conjugated antibodies against CD73, PreCP-CD105 and with phycoerythrin (PE)-conjugated antibodies against CD44 (BD, CA, USA). To assess their ability to differentiate into three different lineages, the hADMSCs were grown in osteogenic-, adipogenic- and chondrogenic- inducting medium for 20 days. Subsequently, the alizarin red staining, oil red O staining and Alcian blue staining were performed to confirm osteocytes, chondrocytes, and adipocytes differentiation, respectively.

### Extraction and characterization of exosomes derived from hADMSCs

The hADMSCs (passage 3–5) at 70–80% confluence was washed with PBS and cultured in FBS-free medium for 48 h. Then the cell supernatants were collected. Exosomes were purified by gradient ultracentrifugation as described previously [27]. Briefly, the centrifugal forces were set on a tabletop centrifuge at 1000 *g* and 10,000 *g* for 10 min and 30 min, respectively. Then, the cell supernatant was filtered through a 0.22 μm sterile filter (Millipore, CA, USA) and centrifugated by the Beckman Coulter Optima™XPN supercentrifuge at 10000 *g* for 70 min, discarding the cell supernatant and resuspend the exosome‐containing pellet in PBS. This centrifugation procedure was repeated twice. 100 μL of PBS was applied to resuspend the final exosomes and stored at − 80 °C. The ultrastructure of hADMSCs-Exo was detected by Transmission Electron Microscope (TEM, Hitachi). The size and concentration distribution of hADMSCs-Exo were characterized by nanoparticle tracking analysis (NTA; NanoFCM N30E), which was high-sensitivity flow cytometry for nanoparticle analysis. The hADMSCs-Exo were labeled using PKH26 Red Fluorescent Cell Linker Kits according to the manufacturer’s instructions (Sigma, USA). Surface exosomal makers such as CD63, CD81 and TSG101 were detected by western blotting (Abcam, USA). The hADMSCs-Exo proteins were quantitatively measured using bicinchoninic acid assay kits (Beyotime, China) according to the manufacturer’s manual.

### Activation of LX-2 cells and treatment with hADMSCs-Exo

Human hepatic stellate cells (LX-2 cells) were purchased from Shanghai Mcellbank Biotechnology Co., Ltd, China, and cultured maintained as described in protocol from the provider. 10 ng/mL TGF‐β1 (Peprotech, 100-21, USA) was added to induce activation of LX-2 cells for 24 h. Activated LX-2 cells were then incubated with different concentrations of hADMSCs-Exo (0.075, 0.15 and 0.3 μg/μL). The same volume of PBS was added to the control group. The cells were examined after 24 h incubation.

### In vitro cell viability

To investigate the cytotoxicity of hADMSCs-Exo, LX-2 cells were seeded at a density of 5000 cells/well in 96-well plates and stabilized for 24 h at 37 ℃ in a humidified 5% CO2 atmosphere. LX-2 cells were activated by TGF‐β1 and incubated for 24 h. Then the cells were incubated with hADMSCs-Exo for 24 h, 48 h or 72 h, and the viability of LX-2 cells in the presence of different concentrations (0, 0.01, 0.05, 0.1, 0.2 and 0.3 μg/μL) of exosomes was quantified by CCK-8 assays (Cat# CK04-01; Dojindo, Kumamoto, Japan). The optical density (OD 450 nm) values were measured by a microplate reader.

### Proliferation, cell cycle analysis and apoptosis detection

Cells were seeded in 6-well plates, treated the next day with hADMSCs-Exo (0.075, 0.15 and 0.3 μg/μL) or an equivalent volume of PBS and then harvested at 24 h. To measure the proliferation of cells, the Cell Light EdU DNA imaging kit (EdU-Click 488, sigma) was used for EdU incorporation experiments according to the kit instructions. The ratio of EdU-stained cells (with green fluorescence) to Hoechst-stained cells (with blue fluorescence) was used to evaluate the cell proliferation activity. Flow cytometry was used for cell cycle and apoptosis assays. Cell Cycle Analysis Kit (KeyGENEBioTECH, KGA511, China) was used for cell cycle analysis and the Annexin V-FITC & PI Cell Apoptosis Analysis Kit (BD, 550911, USA) was used for apoptosis assays. The data were analyzed using FLOWJO software.

### Immunofluorescence staining

When activated HSCs reached 60–70% confluency on 24-well plates, they were cultured with hADMSCs-Exo for 24 h. Next, HSCs were incubated with 4% paraformaldehyde (PFA) for 15 min and then incubated with 5% goat serum (Proteintech, China) for 1 h. Cells were incubated with a primary antibody (anti-α-SMA (Proteintech, 1:300 dilution, China), anti-Fibronectin (Proteintech, 1:300 dilution, China) or anti-AKT (CST, 1:400 dilution) overnight at 4 °C, followed by incubation with Alexa Fluor 488-conjugated goat anti-rabbit IgG (Invitrogen, A-11008) or Alexa Fluor 594-conjugated goat anti-mouse IgG (Invitrogen, A-11005) for 1 h. The nuclei were labeled with DAPI (bioshop, USA). Fluorescent images were captured using Laser Scanning Microscopy (LSM) (Zeiss, LSM 900&Airyscan, Germany).

### RNA extraction and quantitative real-time polymerase chain reaction (RT–PCR)

Total RNA from fresh frozen tissues and cells was isolated using Trizol reagent (Invitrogen, NY, USA) according to the manufacturer’s instructions. RNA was quantified using a NanoDrop (Thermo Fisher Scientific, MA, USA). Subsequently, 1 μg of total RNA was used to produce cDNA using PrimeScript RT reagent (Takara, Kusatsu, Japan), and qRT–PCR was performed using TB Green™ Premix Ex Taq™ II (Takara, Japan) in the CFX96 Real-time System (Bio-Rad). mRNA expression was calculated via the standard 2 − ΔΔCtmethod. Each sample was analyzed in triplicate. The PCR primers utilized in this study were showed in Additional file 1: Table S1.

### Western blot analysis

The RIPA lysis buffer (Solarbio, Beijing, China) was used to conduct the purification of the protein of the hADMSCs-Exo, cells and tissues. Total proteins were extracted and detected by BCA, then separated by 8–12% SDS‐PAGE and transferred to membranes (Millipore Corp, Billerica, USA). After blocking, membranes were incubated with primary antibodies and horseradish peroxidase‐conjugated secondary antibodies. Signals were detected and quantified with ChemiDoc XRS + system using Image Lab software (Bio‐Rad, Hercules, CA, USA) with the Immobilon Western Chemiluminescent HRP Substrate (Millipore). Antibodies utilized in this study are listed in Additional file 1: Table S2.

### Experimental animals

Male mice (C57BL/6 J, 20–25 g, 6 weeks of age) were purchased from Beijing Weitong Lihua Experimental Animal Technology Co. Ltd. All procedures involving animals were performed in accordance with the National Institutes of Health Guide for the Care and Use of Laboratory Animals and were approved by the Animal Ethic Committee of Sichuan Provincial People’s Hospital (Approval number: 2021448). Procedures for CCl4–induced liver fibrosis were as detailed elsewhere [28]. After adaptive feeding for a week, animals were randomly divided into liver fibrosis group modeling and normal group, with administered CCl4 (1 mL/kg intraperitoneally; diluted 1:4 in olive oil) or olive oil vehicle-alone 2× week for 8 weeks, respectively. Then Liver Fibrosis Group (LFG) was sacrificed. Regression group (REG) are treated with 200 μl PBS, each mouse in the hADMSCs group received 1 × 10^6^ hADMSCs in 200 μl PBS, the hADMSCs-Exo group received 200 μL PBS containing 250 μg hADMSCs-Exo, doses were based on published literature[29, 30]. All of the treatments were given intravenously via tail vein injection and carried out twice per week for 4 weeks. Blood was taken from eyeball and immediately centrifuged for serum collection. Liver tissue was excised and snap-frozen in liquid nitrogen.

### Shear wave elastrography (SWE)

After 8 weeks of CCl4 treatment, liver stiffness of all animals was measured by transient elastography (FibroScan; Echo‐Sens, Paris, France). Procedures for SWE were as detailed elsewhere [31]. Ultrasound elastography data were collected prior to the start of treatment (baseline), during treatment (weeks 2) and a final scan prior to sacrifice (weeks 4).

### Liver histopathological evaluation, immunohistochemistry analysis and ELISA

Liver tissues from the same lobe were embedded in paraffin, sectioned at 5 µm. Hematoxylin and eosin (H&E), Masson's trichrome (MT) staining and Sirius red staining were carried out using paraffin‐embedded sections. Liver fibrosis and necro-inflammatory activity were semi-quantitatively evaluated in a blind manner by two independent pathologists and scored according to the Ishak fibrosis scoring system. For MT and Sirius red staining, quantitative analysis was performed using ImageJ at 100× magnification in 5 random fields. For immunohistochemistry (IHC) staining, sections were sectioned and cultured overnight with primary antibodies against α-SMA, Collagen I, Ki67, HNF-α, or Caspase 3 overnight at 4 °C and with secondary antibodies (Abcam) for 1 h at 37 °C. The nickel-diaminobenzidine (DAB) color reagent kit (Proteintech, China) was used for color development. The histology of the different tissues was analyzed microscopically (BX51, Olympus, Tokyo, Japan). Image capturing was performed using DP2-BSW software (Olympus). Choline, phosphorylcholine, phosphatidylcholine, phingomyelin, glycerophosphocholine and Betaine Assay kits were purchased from Shanghai Yanzun Biotechnology Co.Ltd. Procedures for Enzyme-linked immunosorbent assay (ELISA) were as described elsewhere [32].

### Blood biochemical assay

To investigate the liver function test in blood, the isolated serum of each mouse was analyzed for aspartate aminotransferase (AST), alanine aminotransferase (ALT), alkaline phosphatase (ALP). These parameters of the serum were evaluated by ChemOn Inc (Suwon, Korea).

### RNA-seq and data analysis

RNA was isolated from liver of mouse after indicated different treatments using Trizol Reagent (Invitrogen Life Technologies). Three micrograms of RNA were used as input material for the RNA sample preparations. The sequencing library was constructed by using the NEBNext^®^ Ultra RNA Library Prep Kit for Illumina^®^ (NEB England BioLabs), then sequenced on NovaSeq 6000 platform (Illumina). Differentially expressed genes (DEGs) were screened with the adjusted P-value of < 0.05 and |log fold change| of ≥ 1. Heat maps and Gene Expression Enrichment Analysis were generated using Pheatmap and ggplots2 in R. Pathway analysis was performed by using clusterProfler23 package in R.

### LC–MS/MS analysis

A total of 20 mg of each tissue sample was homogenized with 0.6 ml of 2-chlorophenylalanine (4 ppm) containing methanol (− 20◦C), centrifuged, and filtered through a 0.22-μm membrane before analysis on an ultra-performance liquid chromatography (UPLC)-mass spectrometry (MS). LC conditions and MS conditions were as described previously [33]. Data-dependent acquisition was employed to identify metabolites and the unnecessary MS/MS information was removed by dynamic elimination. Metabolite identification was performed by metabolome databases including MassBank (http://www.massbank.jp/index.html), the human metabolome database (http://www.hmdb.ca/), METLIN (http://metlin.scripps.edu/index.php), lipidmaps (https://www.lipidmaps.org/), mzCloud (https://www.mzcloud.org/) and the self-built database of BioNovoGene by Suzhou PANOMIX Biomedical Tech Co., Ltd. Metabolic pathway enrichment analysis was carried out using Metaboanalyst 5.0 (https://www.metaboanalyst.ca/).

### Statistical analysis

All data were used of GraphPad Prism13.0 software, Image J analyses and R 3.6.0 (The R Foundation for Statistical Computing, Vienna, Austria). One-way ANOVA and *t*-test was used for comparison of two or more groups. P < 0.05 was considered to be statistically significant.

## Results

### Characterization of hADMSCs and hADMSCs‑Exo

**Fig. 1:**
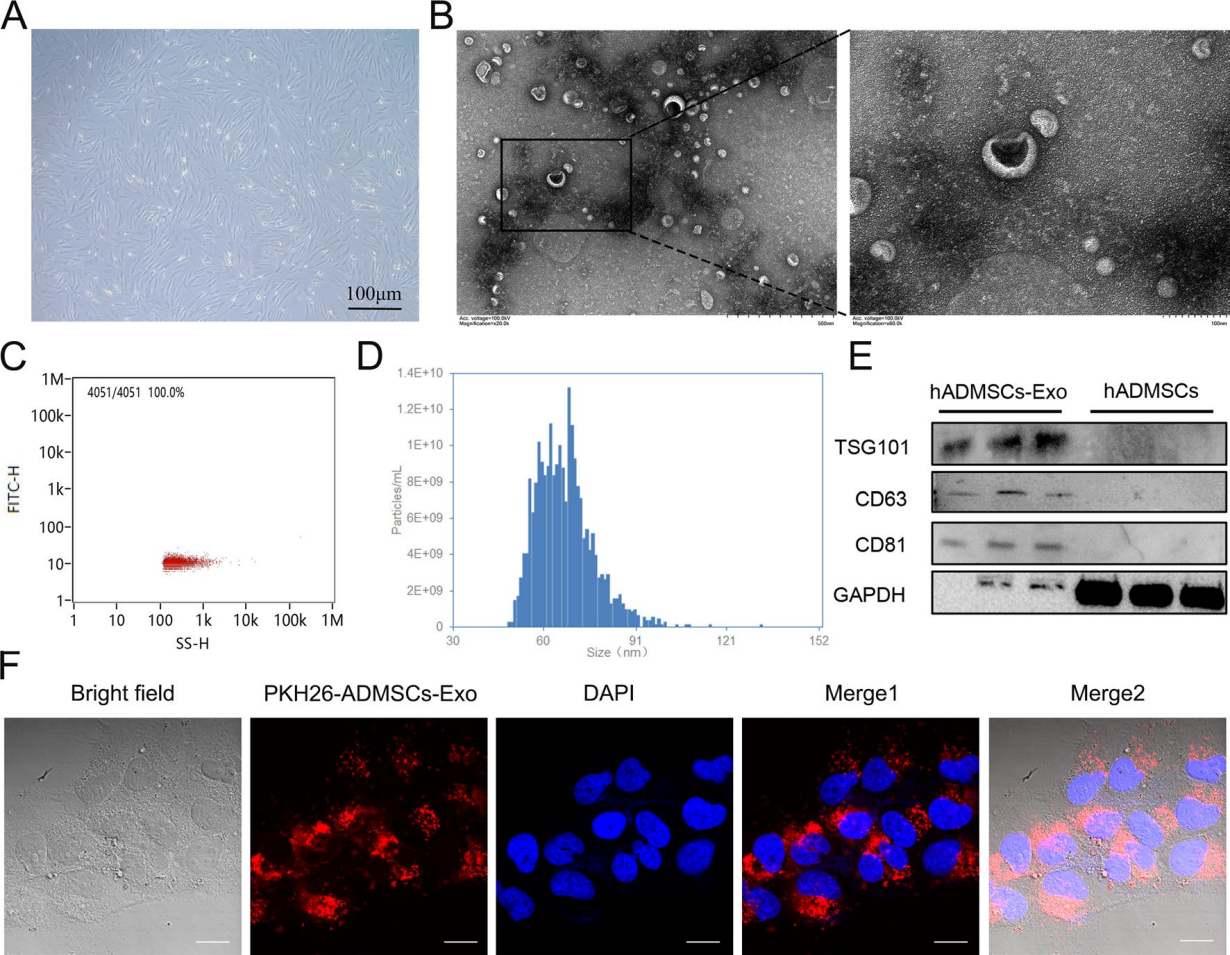
Characterization of and hADMSCs and hADMSCs-Exo. **A** Morphological appearance of cultured hADMSCs (bar = 100 μm). **B** Representative TEM images of hADMSCs-Exo (bar = 500 nm and 100 nm). **C** Flow cytometry for nanoparticle analysis of hADMSCs-Exo. **D** Size distribution measurements of hADMSCs-Exo by NTA under flow conditions. **E** Western blot analysis of TSG101, CD63, CD81 and GAPDH in hADMSCs-Exo and whole-cell lysis of hADMSCs. **F** CLSM images of hADMSCs-Exo labeled with PKH26. Red: PKH26-Exo. Blue: DAPI. Scale bar, 40 μm

In the beginning, primary cells isolated from human adipose tissues underwent identification of immunophenotype and multipotential differentiation ability, for the purpose of providing exosome constantly. The morphology of hADMSCs was monolayer adherent and fibroblast-like spindle-shaped (Fig. 1A). For detecting multilineage differentiation potential of hADMSCs, we evaluated osteogenic, adipogenic and chondrogenic differentiation of hADMSCs by using different inducing conditioned medium respectively. Morphological changes were shown in Additional file 1: Fig. S1A, compact cell layer and intense Alizarin red staining illustrated calcium deposition (bright red spots) of osteogenically differentiated tissues, supporting the osteogenic differentiation ability of hADMSCs. Lipid droplets (dark red spots) formed during adipogenic differentiation of hADMSCs were visualized by Oil Red O staining. After 1 month of chondrogenic differentiation in culture, we observed clear cartilage formation, and production of proteoglycan (blue circle) by Alcian blue staining, suggesting the multi-lineage differentiation ability of hADMSCs. In addition, to better understand the immunophenotype of hADMSCs, we examined expression of characteristic stem-cell surface markers (CD44, CD73, CD90, and CD105) by utilizing flow cytometry. The result showed positive rate for CD44, CD73, CD90 and CD105 of hADMSCs were 85.1%, 99.3%, 99.6% and 98.7% respectively (Additional file 1: Fig. S1B). Collectively, these data identified the osteogenic, chondrogenic, and adipogenic differentiation hADMSCs isolated from human adipose tissues, as well as their immune phenotypes.

Next, exosomes derived from hADMSCs underwent evaluation on morphology and content. The ultra-microstructure of purified hADMSCs-Exo was elliptical vesicle structures with the similar size of ~ 100 nm (Fig. 1B). Result of NTA-based characterization showed that the size of hADMSC-Exo was concentrated in the range of 50–100 nm (average size = 76.14 nm), while the particles of 30–150 nm accounted for 96.66% of the total particles with a concentration of 4.11E + 11 Particles/mL (Fig. 1C, D). Through detecting exosomal biomarkers by western blot assay, we observed strong expression of TSG101, CD63 and CD81 in hADMSCs-Exo rather than hADMSCs lysates (Fig. 1E). Furthermore, hADMSCs-Exo were labeled with PKH26 (red) and cocultured with LX-2 cells. Labeled hADMSCs-Exo were engulfed by LX-2 cells and were distributed around their nucleus (Fig. 1F).

### hADMSCs-Exo inhibits hepatic stellate cell proliferation by arresting cell cycle and inducing apoptosis

**Fig. 2:**
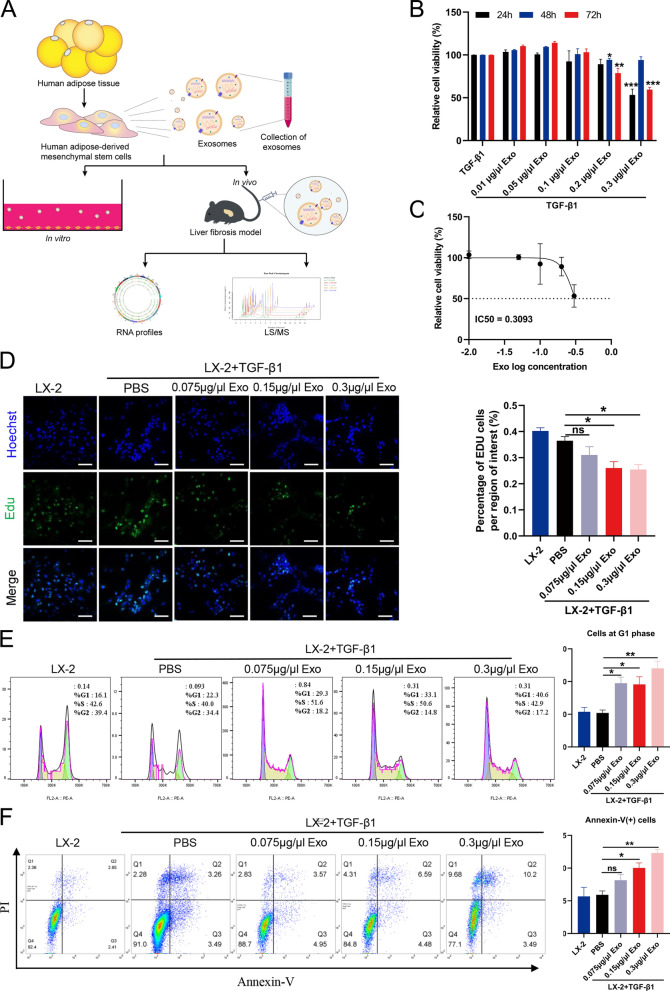
hADMSCs-Exo inhibits hepatic stellate cell proliferation by impeding cell cycle progression and inducing apoptosis. **A** A schematic representation of the experimental design. **B** In vitro cell viabilities of activated LX-2 cells incubated with hADMSCs-Exo at the indicated concentration in the presence of TGF-β1 (10 ng/ml) for 24 h, 48 h or 72 h, (n = 5). **C** IC50 were analysed in activated LX-2 cells exposed to hADMSCs-Exo for 24 h. **D** Edu assay showed that HSCs proliferation was suppressed by hADMSCs-Exo in a concentration-dependent manner. EdU% is used as an approximation for proliferation rate. **E** Cell cycle analysis showed an increase in the sub-G1 subpopulation and cell cycle arrest after hADMSCs-Exo. **F** The quantitative analysis of the apoptosis was performed using the Annexin-FITC staining based flow cytometry. Exo, exosome. Data are presented as means with SEM (n = 3 independent experiments). ns, not significant, *p < 0.05, **p < 0.01 and ***p < 0.001

To investigate the repairment function of hADMSCs-Exo on fibrosis, we designed both in vitro and in vivo experiments as illustrated in Fig. 2A. Considering that TGF-β1-regulated hepatic stellate cells (HSCs) are responsible for liver fibrosis, we constructed the fibrotic cell model by treating LX-2 cells with TGF-β1. Morphologic change and expression of both profibrotic and EMT-related proteins in LX-2 cells were examined at different time points (12 h, 24 h, 48 h and 72 h). As shown in Additional file 1: Fig. S2A, compared to that quiescent HSCs were long spindle shape, LX-2 cells at 12 h after TGF-β1 treatment formed pseudopodia with elongated synapses. At the timepoint of 24 h after activation, LX-2 cells exhibited clearer filopodia, lamellipodia, and polygonal shapes. The cells displayed disordered arrangment, tight junction, aggregative growth, and cellular protrusions obviously at 48 h or 72 h. Western blot results showed EMT appeared in LX-2 cells after 12 h, and profibrogenic markers (α-SMA) could be stably expressed at 24 h (Additional file 1: Fig. S2B). Based on these observations, the time point of 24 h after TGF-β1 stimulation was used for constructing fibrotic cell model in the subsequent experiments.

To determine the effect of hADMSCs-Exo on activated HSCs, we evaluated cell proliferation after being treated with hADMSCs-Exo. As showed in Fig. 2B, proliferation of pro-fibrotic HSCs was inhibited by hADMSCs-Exo in a concentration-dependent fashion. High concentration of hADMSCs-Exo (0.3 μg/μl) decreased cell survival rate by half, whereas low‐dose hADMSCs-Exo (range from 0 to 0.1 μg/μL) did not alter proliferation of activated HSCs by CCK8 assay (Fig. 2C). Dose-dependent inhibiting effect of hADMSCs-Exo on HSCs were confirmed by Edu proliferation assay, which showed an augmented HSCs proliferation in hADMSCs-Exo-treated groups (Fig. 2D). In addition, we observed that hADMSCs-Exo arrested cell cycle arrest in G1 phase of cell cycle, a small, non-significant increase in cells in S phase, and gradually declined at the G2 phase (Fig. 2E). Similarly, we also tested the impact of hADMSCs-Exo on apoptosis of activated HSCs. A concentration-dependent increase in apoptosis occurred in activated HSCs after 24 h of exposure to hADMSCs-Exo (Fig. 2F).

### hADMSCs-Exo internalized by LX-2 cells exhibit antifibrotic and anti-EMT effect in vitro

**Fig. 3:**
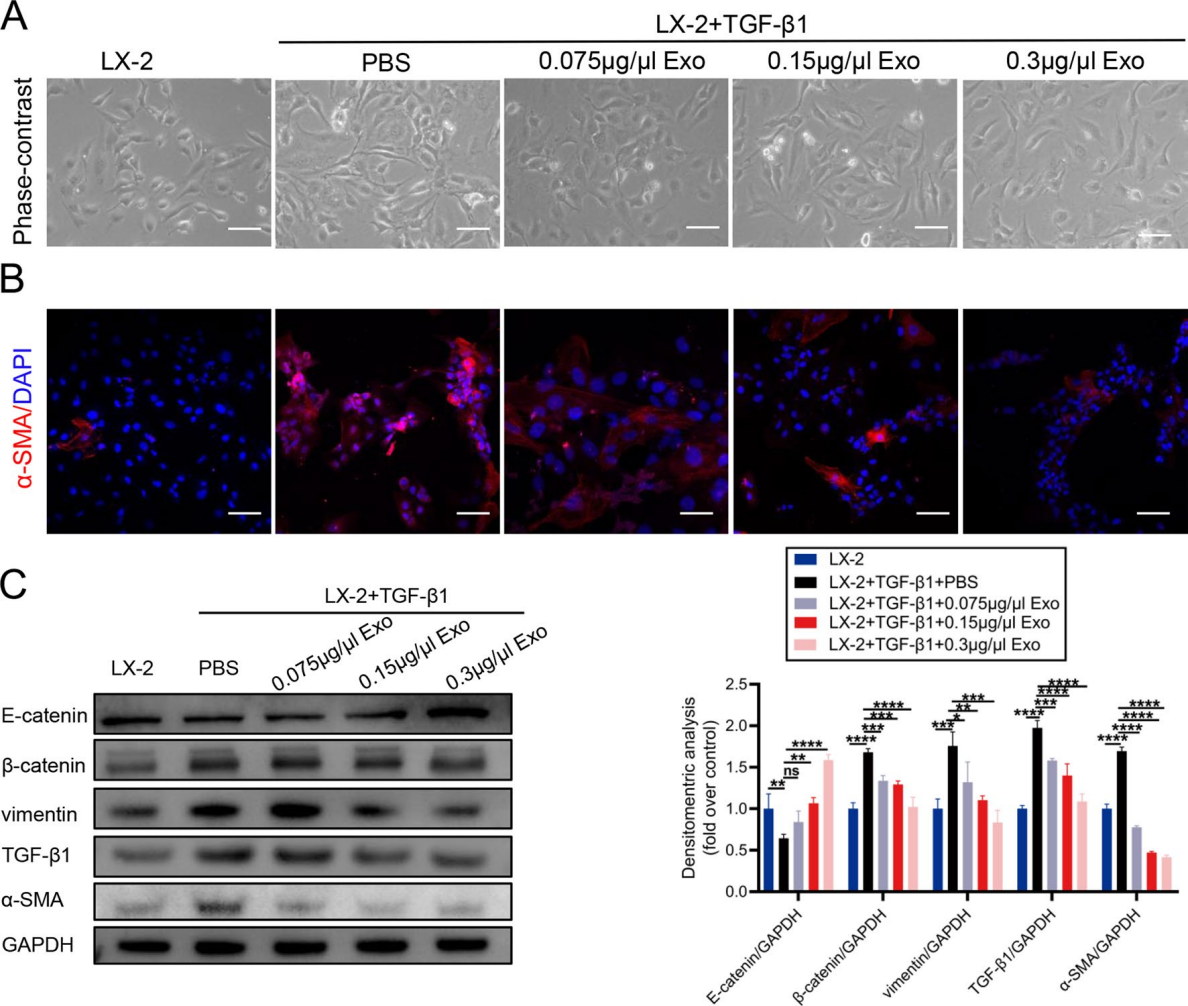
hADMSCs-Exo internalized by LX2 cells exhibit antifibrotic and anti-EMT effect in vitro. **A** Representative phase-contrast images of quiescent HSCs and aHSCs were treated with hADMSCs-Exo at the indicated concentration in the presence of TGF-β1 (10 ng/ml). Analyses were conducted 24 h after the indicated treatments. Scale bars, 100 mm. **B** Representative immunofluorescence images of α-SMA (red) and DAPI (blue) of aHSCs were treated with hADMSCs-Exo. Scale bars, 100 mm. **C** Western blot analysis of LX-2 cell incubated with hADMSCs-Exo for 24 h in the presence of TGF-β1 (10 ng/ml). Data expressed as the mean ± SEM (n = 3 independent experiments). ns, not significant, *p < 0.05, **p < 0.01, ***p < 0.001 and ****p < 0.0001

Subsequently, we explored the mechanism involved in influence of hADMSCs-Exo on activated HSCs in a LX-2 and hADMSCs co-culture system. As shown in Additional file 1: Fig. S3, hADMSCs significantly inhibited proliferation of activated LX-2 cells and blocked expression of α-SMA. To investigate whether the inhibitory effect on LX-2 is mediated exosome, we treated activated LX-2 cells with purified hADMSCs-Exo. As showed in Fig. 3A, our results showed that compared to original LX-2 cells, TGF-β1 activated LX-2 cells formed filopodia- and lamellipodia-like extensions. These cell protrusions shrunk gradually when being incubated with hADMSCs-Exo, or even completely disappeared after being incubating with high concentration of hADMSCs-Exo. Concurrently, the immunofluorescence assay results revealed that when aHSCs were treated with hADMSCs-Exo, the fluorescence signals of α-SMA significantly diminished in a dose-dependent manner, indicating excellent antifibrotic effect of hADMSCs-Exo (Fig. 3B). In addition, antifibrotic and anti-EMT effect of hADMSCs-Exo were evaluated by western blotting. After being educated with hADMSCs-Exo, ECM of LX-2 cells was markedly degraded, performed with downregulated collagen I and α-SMA expression. Reduced TGF-β1, vimentin and β-catenin whereas increased E-catenin suggested that EMT process was substantially inhibited as well. These results suggested that hADMSCs-Exo could inactivate the activated HSCs through EMT inhibition and ECM degradation.

### hADMSCs-Exo could alleviate CCl_4_-induced mice liver fibrosis in vivo

For further verification, therapeutic effect of hADMSCs-Exo and hADMSCs were examined in fibrotic mice, and the samples were analyzed by RNA-seq and LC–MS/MS. Before that, we used CCl_4_ to establish liver fibrosis mouse model, which is verified by gross inspection and histological analysis (Additional file 1: Fig. S4A, B). We investigated the organ distribution of hADMSCs-Exo in fibrotic mice especially in their liver tissue. PKH26-labeled hADMSCs-Exo were injected through tail vein into the models, internal distribution of them were traced using a NIRF fluorescence imaging system. As expected, higher fluorescence signals of hADMSCs-Exo in models were detected in fibrotic liver tissue earlier than in other organs (Additional file 1: Fig. S5A, B).

**Fig. 4:**
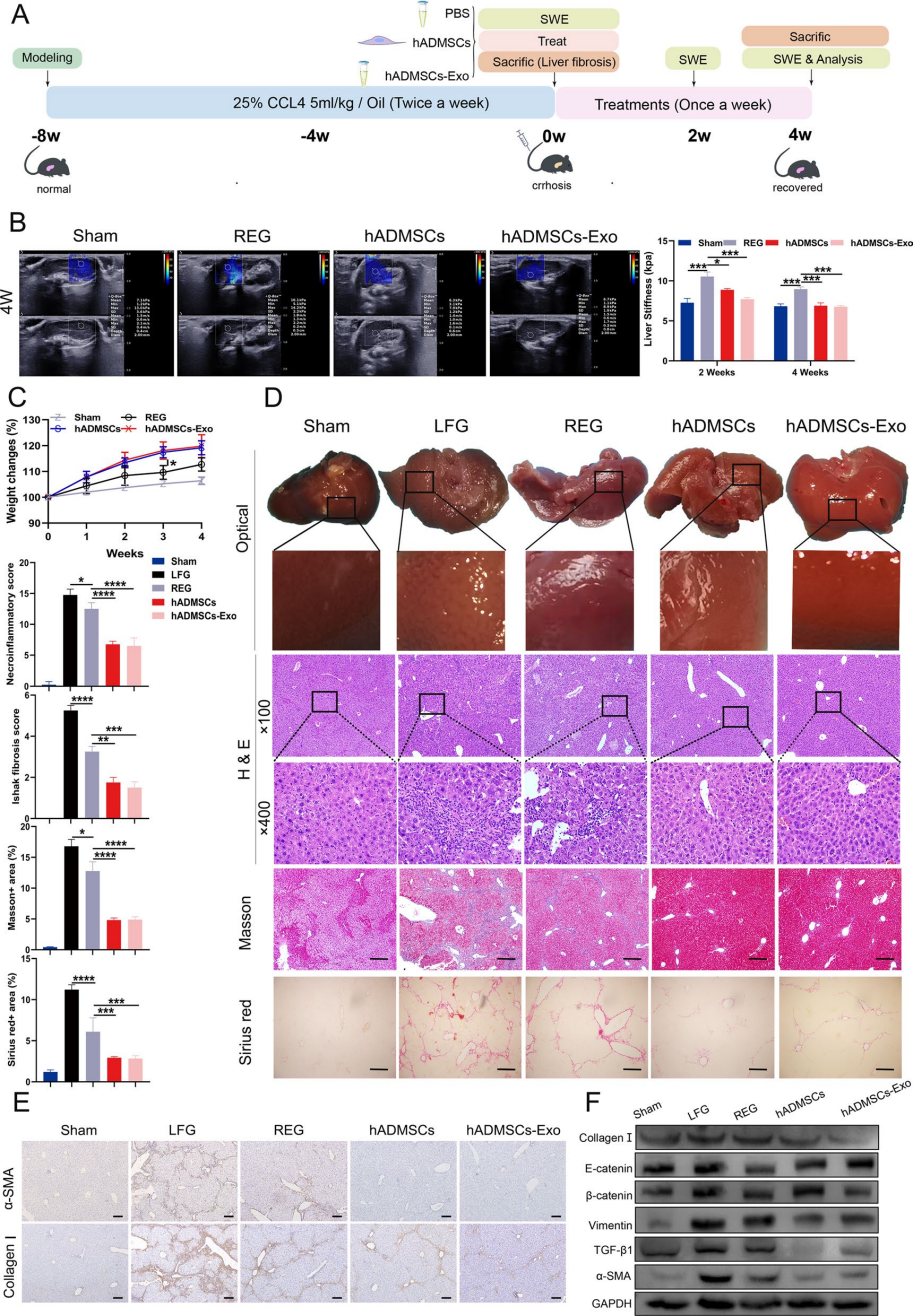
hADMSCs-Exo could alleviate CCl_4_-induced mice liver fibrosis in vivo **A** Diagram of experimental scheme. **B** Elastograms obtained using shear wave elastrography (reliable images were obtained when uniform colour filled > 90% of the sampling area) and liver stiffness quantification. **C** Body weight changes during treatment. **D** Representative photographs of lives from mice in each group and histopathological images of liver sections were evaluated using H&E, Masson trichrome staining and Sirius red staining in animals with CCl_4_-induced cirrhosis. Liver histopathology grading was evaluated by necro-inflammatory scoring and Ishak (modified Knodell) scoring system after treatment. **E** Images of immunohistochemistry staining of extracellular matrix (ECM) proteins (collagen I and α-SMA). **F** The protein levels of ECM and EMT-related proteins in the liver tissues of the mice with different treatments. SWE, shear wave elastrography. LFG, liver fibrosis group, REG, regression group. Data are expressed as mean ± SEM (n = 6), ns, not significant, *p < 0.05, **p < 0.01, ***p < 0.001 and ****p < 0.0001

Based on the fibrotic mice model, we assessed the therapeutic potential of hADMSCs-Exo as depicted in Fig. 4A. SWE verified the success of modeling, and showed a significant increase in mice liver stiffness in the other groups compared with sham group (Additional file 1: Fig. S6). We next assess liver stiffness measurement (LSM) by SWE after 2 weeks and 4 weeks of treatment. Compared to normal liver tissue, liver stiffness value was significantly elevated in regression group (REG). Hepatic fibrosis wasn’t completely repaired after 2 weeks of hADMSCs-Exo treatment. Liver matrix stiffness significantly declined to normality after 4 weeks of treatments or hADMSCs or hADMSCs-Exo, demonstrating a repaired status compared to REG group (Fig. 4B, Additional file 1: Fig. S7). In addition, the body weight of hADMSCs group and hADMSCs-Exo group increased faster compared with sham group and REG, demonstrating recovered status under treatment with hADMSCs or hADMSCs-Exo (Fig. 4C).

At four weeks post to treatment, liver tissues were collected for evaluating effectiveness of hADMSCs-Exo in recovering liver injury, hepatic inflammation and fibrotic degree. As shown in Fig. 4D, fibrotic liver manifested with hard texture, rough surface with brownish color and nodule formation, indicating a serious injured condition. Intriguingly, liver in the hADMSCs-Exo group showed clear morphological restoration along with a smooth surface and bright red color in macroscopic images. The results showed that hADMSCs-Exo and hADMSCs groups showed a reduction of hepatocyte piecemeal, confluent necrosis, ductal proliferation and infiltration of immune cells in histological examinations and their staging score of liver fibrosis, which was dramatically lower than that in LFG and REG (1.5, 1.75, 3.25, 5.25, p < 0.05). Masson’s trichrome and Sirius Red staining were performed to indicate collagen deposition in liver sections. The collagen-stained area of the hADMSCs-Exo group was 2.6 times lower than that of the REG and 3.4 times lower than that of the LFG. These results were consistent with those of α-SMA and collagen I staining, showing that a remarkable reduction was found in the α-SMA and collagen I stained areas of the hADMSCs-Exo-treated group (Fig. 4E). Furthermore, Western blot experiments verified the above result and confirmed anti-EMT effect of hADMSCs-Exo (Fig. 4F). Thus, these data substantiated that systemic administration of hADMSCs-Exo could histologically and functionally alleviate liver fibrosis in CCl_4_-induced mice liver fibrosis.

### hADMSCs-Exo treatment improves liver function and regeneration, reduces liver inflammation and apoptosis

**Fig. 5:**
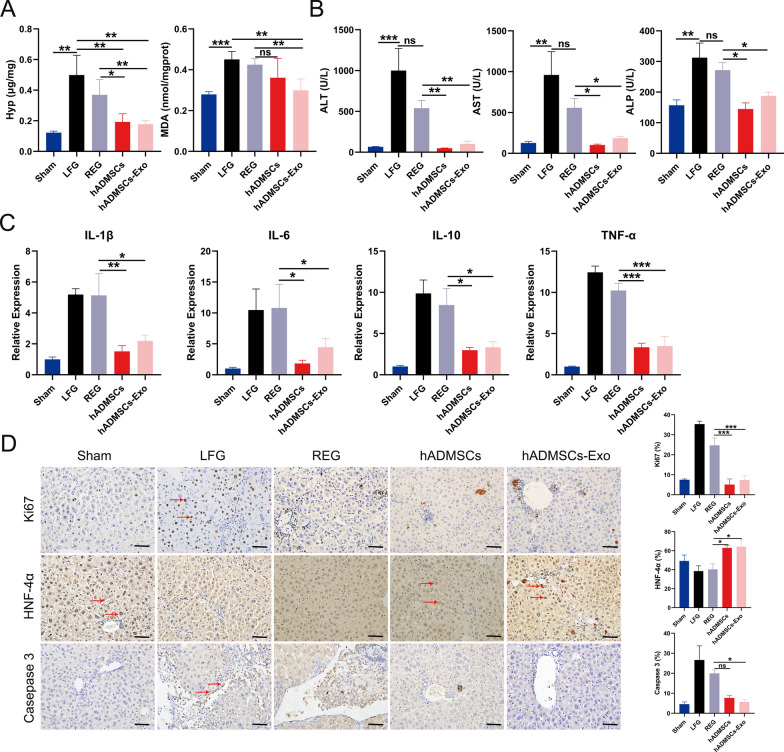
hADMSCs-Exo treatment improves liver function and regeneration, reduces liver inflammation and apoptosis. **A** The Hyp and MDA levels of normal mice (Sham), fbrotic mouse model and the mice injected with PBS, hADMSCs or hADMSCs-Exo were measured with corresponding test kit **B** Serum levels of AST, ALT, ALP in different groups. **C** The relative inflammatory gene expression for IL-1β, IL-6, IL-10 and TNF-α. **D** Immunohistochemical staining was performed to detect the protein expressions of Ki67, HNF-4α and caspase 3 in the injured liver of mice treated with hADMSCs and hADMSCs-Exo. Scale bar, 40 μm. AST, aspartate aminotransferase, ALT, alanine aminotransferase, ALP, alkaline phosphatease. LFG, liver fibrosis group, REG, regression group. Data are expressed as mean ± SEM (n = 4), ns, not significant, *p < 0.05, **p < 0.01 and ***p < 0.001

Next, we examined levels of hydroxyproline (Hyp) and malondialdehyde (MDA) in liver tissues, which indicate the presence of liver collagen deposition and lipid peroxidation changes, reflecting the degree of hepatic oxidative stress, inflammation and hepatocellular injury, respectively. Treatment with hADMSCs reduced Hyp and MDA accumulation in comparison with the LFG and REG, which was even lower in hADMSCs-Exo-treated group (Fig. 5A), suggesting a superior therapeutic effect of hADMSCs-Exo than hADMSCs in regulating collagen deposition and oxidative stress. Similar results were obtained when performing serum biochemical tests and quantifying inflammatory cytokines. Serum levels of ALT, AST and ALP were significantly suppressed in the hADMSCs and hADMSCs-Exo-treated mice when compared with LFG and REG (Fig. 5B). Furthermore, expression of inflammatory cytokines including Interleukin-1β (IL-1β), Interleukin-6 (IL-6), Interleukin-10 (IL-10) and tumor necrosis factor-α (TNF-α) were significantly decreased in livers of hADMSCs-Exo group compared with those of the LFG and REG, demonstrating effective anti-inflammatory effects of hADMSCs-Exo in liver fibrosis mice.

To evaluate whether hADMSCs-Exo treatment could reduce hepatocellular injury and apoptosis, improve liver regeneration, we performed IHC to quantify Ki-67, caspase 3 and HNF-4α. As shown in Fig. 5D, the percentage of Ki-67^+^ cells in hADMSCs (5.09%) and hADMSCs-Exo (7.35%) treated groups was decreased significantly when compared to the LFG (35.31%) and REG (28.64%). The percentage of HNF-4α^+^ cells in hADMSCs-Exo (64.27%) group increased significantly when compared with the LFG (38.50%) and REG (40.38%). hADMSCs-Exo exhibited decreased hepatocyte death characterized by remarkably decreased caspase-3 expression, indicating that hADMSCs-Exo reduced hepatocellular injury and apoptosis, and recovered from CCl_4_-induced liver damage.

### hADMSCs-Exo inhibited HSCs activation and liver fibrosis through PI3K/AKT/mTOR signaling pathway

**Fig. 6:**
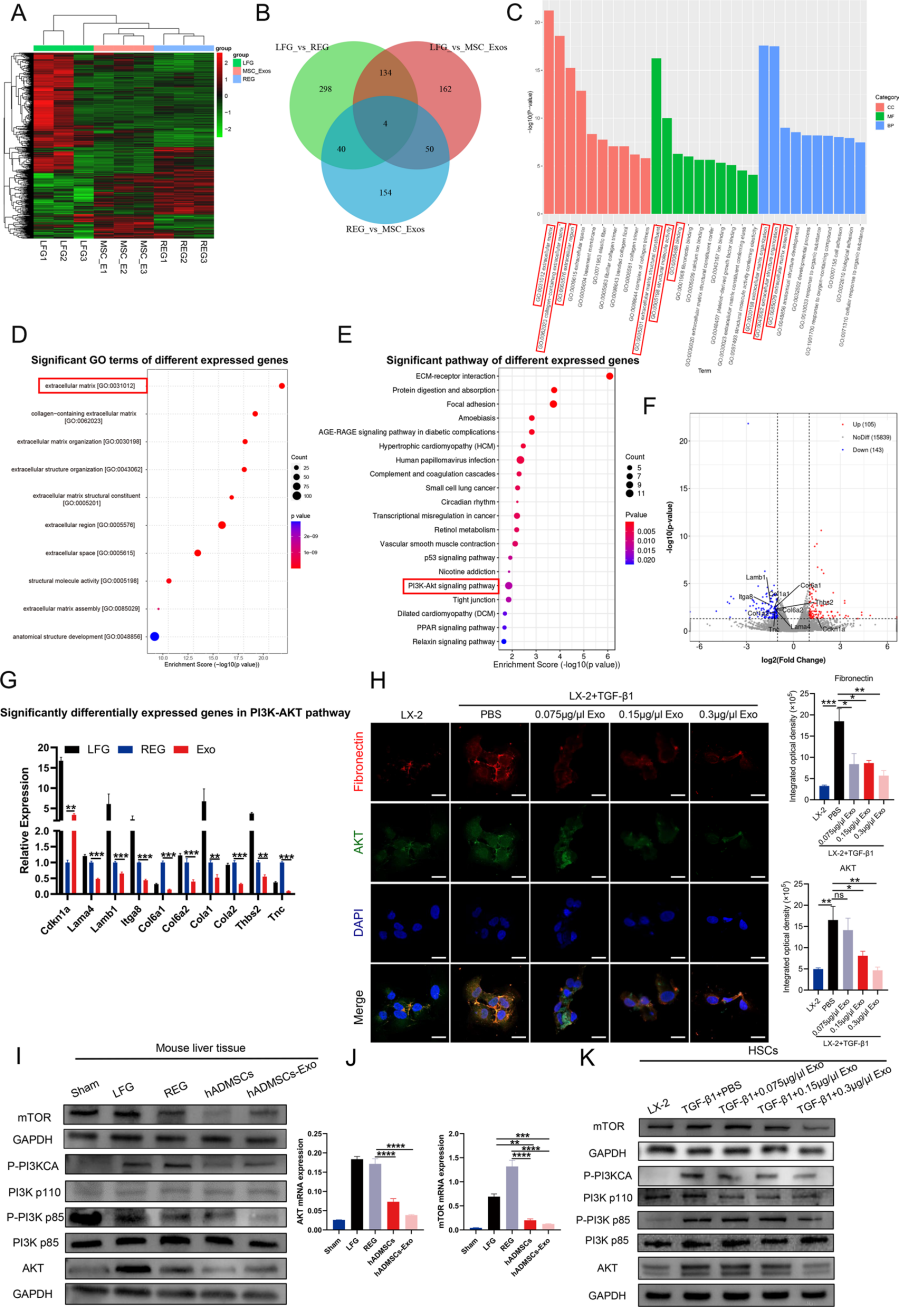
hADMSCs-Exo inhibited HSCs activation and liver fibrosis through PI3K/AKT/mTOR signaling pathway. **A** Heatmap of differential expression analysis of RNA-sequencing data from LFG, REG and hADMSCs-Exo. **B** Venn diagram of LFG vs REG, LFG vs hADMSCs-Exo and REG vs hADMSCs-Exo. **C** GO analysis results of the DEGs between REG and hADMSCs-Exo, including cell component (CC), molecular function (MF) and biological process (BP). Red represents CC, green represents MF, blue represents BP. **D** The top 10 significant GO terms of DEGs. **E** KEGG analysis of significant pathway of DEGs. **F** Volcano plot and significantly DEGs in PI3K/AKT pathway. Red represents up-regulated, blue represents down-regulated genes. **G** The mRNA levels of significantly DEGs in PI3K/AKT pathway were determined by qRT–PCR. **H** Fibronectin and AKT protein levels in LX-2 or treated with pbs, hADMSCs-Exo in the presence of TGF-β1 (10 ng/ml) for 24 h were measured by immunofluorescence staining. **I** Western blot assay for AKT, mTOR and phosphorylated and total PI3K p85 and PI3K p110 in mouse liver issue. **J** Quantification of AKT and mTOR by qRT–PCR. **K** The expression level of AKT, mTOR and phosphorylated and total PI3K p85 and PI3K p110 after HSCs were incubated with hADMSCs-Exo. LFG, liver fibrosis group, REG, regression group. Data are presented as means with SEM (n = 3 independent experiments). ns, not significant, *p < 0.05, **p < 0.01, ***p < 0.001 and ****p < 0.0001

To unearth the molecular mechanism involved in regulation of hADMSCs-Exo in liver fibrosis, we compared transcriptomic profiles between the LFG, REG, hADMSCs and hADMSCs-Exo group as presented in Fig. 6A. Among these differentially expressed genes (DEGs), we observed a partial intersection of 264 unique DEGs between the hADMSCs-Exo group versus REG, and 350 unique DEGs between the hADMSCs-Exo group versus LFG (Fig. 6B). Gene Ontology (GO) and Kyoto Encyclopedia of Genes and Genomes (KEGG) pathway analyses were performed using these unique DEGs (Fig. 6C and Additional file 1: Fig. S7A, B). In GO terms, the major differentially expressed pathways involved are ECM components and related pathways, which is consistent with our results showing that hADMSCs-Exo regulated ECM remodeling in HSCs. Phosphatidylinositol 3- kinase/protein kinase B (PI3K/AKT) was identified as the top-ranked signaling pathway (Fig. 6D, E, Additional file 1: Fig. S7C, D). A batch of factors in PI3K/AKT signal pathway was downregulated by hADMSCs-Exo, including *Cdkn1a*, down-regulated genes: *Col6a1, Lamb1, Itga8, Tnc, Lama4, Col6a2, Thbs2, Col1a1, Col1a2* (Fig. 6F, Additional file 1: Table S3). Reduced expression of these genes by hADMSCs-Exo group was confirmed by qRT–PCR (Fig. 6G). Through immunofluorescence assay, we observed that AKT protein expression was remarkably reduced following hADMSCs-Exo treatment, while fibronectin protein expression was notably influenced (Fig. 6H). This result suggests that hADMSCs-Exo may downregulate nuclear expression of fibrosis-related genes by inhibiting PI3K-AKT signaling pathway, inhibiting the formation of ECM to ameliorate the progression of liver fibrosis.

To determine whether PI3K/AKT signaling pathway mediates efficacy of hADMSCs-Exo, we analyzed activities of AKT and mTOR expression in activated HSCs and liver tissues. In consistent with transcriptomic result, western blot analysis showed that phosphorylation levels of PI3K were significantly inhibited, a statistically significant decrease was accordingly observed in the AKT and mTOR expressions in vivo (Fig. 6I). qRT–PCR also confirmed decreased mRNA expression of AKT, mTOR in hADMSCs-Exo group ((Fig. 6J, Additional file 1: Fig. S8A). Similarly, PI3K/AKT/mTOR signaling was also inhibited in HSCs treated with hADMSCs-Exo in a concentration-dependent manner (Fig. 6K, Additional file 1: Fig. S8B). These results confirmed that PI3K/AKT/mTOR signaling pathway is participated in function of hADMSCs-Exo.

### hADMSCs-Exo inhibits liver fibrosis by regulating choline metabolism

**Fig. 7:**
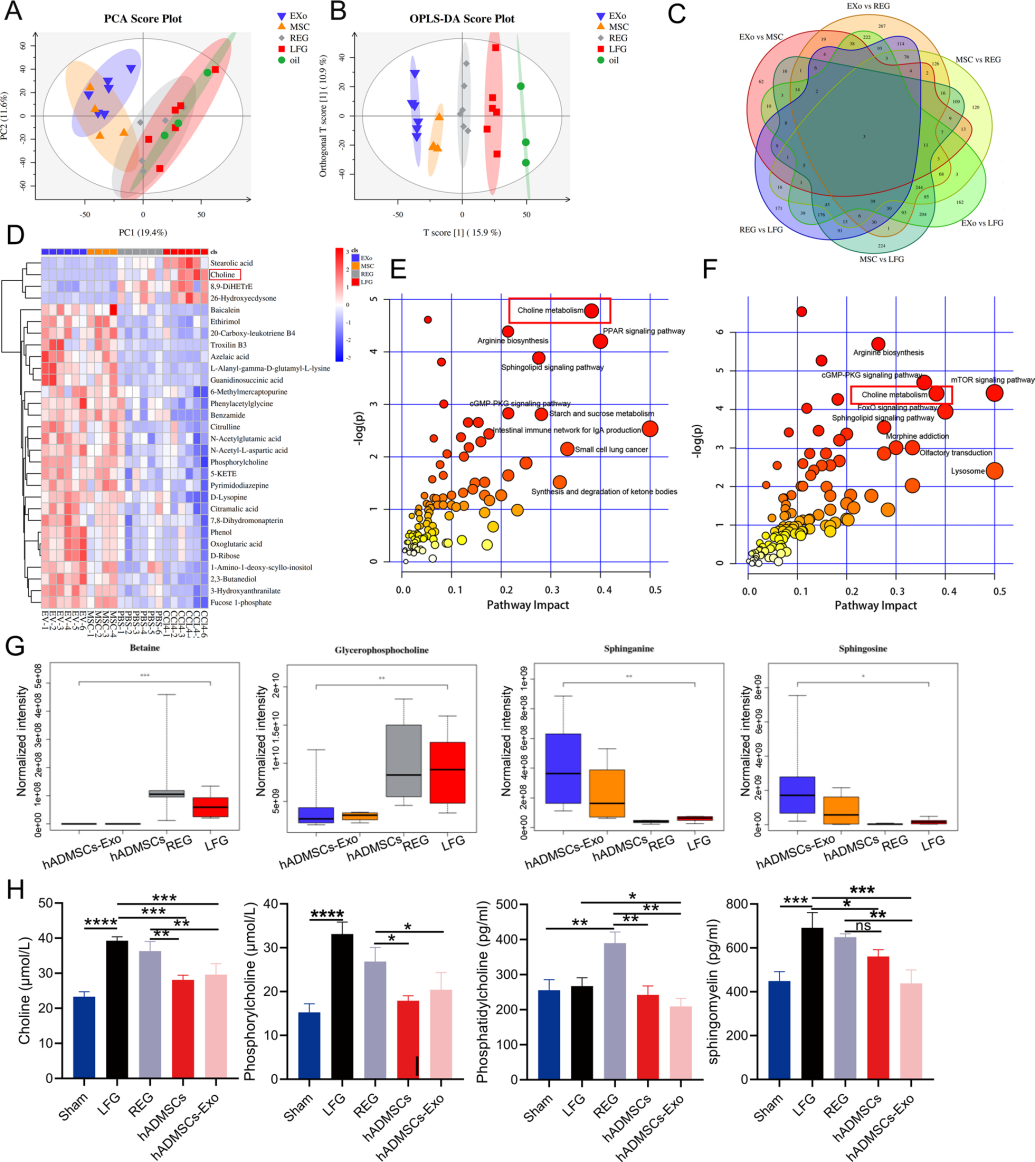
hADMSCs-Exo inhibits liver fibrosis by regulating choline metabolism. **A** Score plots from the PCA model derived from the UPLC-MS profile of liver obtained from mice in different groups. **B** Score plots from the OPLS-DA model from metabolic profiles of different groups. **C** Venn diagram of differential metabolites between LFG, REG, hADMSCs and hADMSCs-Exo-treated group. **D** The hierarchical clustering heatmap of the top 30 metabolites between different groups. **E** Summary of pathway analysis of differential metabolites between REG and hADMSCs-Exo-treated group. **F** Summary of pathway analysis of differential metabolites between LFG and hADMSCs-Exo-treated group. **G** The quantitative analysis of the key metabolites in choline metabolism based on metabolic profiles. **H** Quantitative analyses of the key metabolites in choline metabolism by ELISA. LFG, liver fibrosis group, REG, regression group. Data expressed as the mean ± SEM. ns, not significant, *p < 0.05, **p < 0.01, ***p < 0.001 and ****p < 0.0001

To unearth mechanisms underlying hADMSCs-Exo-mediated metabolic activities in inhibiting liver fibrosis, we quantified and compared metabolites in liver with hADMSCs-Exo treatment by LC/MS technology. Through principal components analysis (PCA), metabolites of mouse liver tissue in each group were obviously discriminated to five different groups (Fig. 7A), and Orthogonal partial least squares discriminant analysis (OPLS-DA) revealed a clear and statistically significant separation among each group (OPLS-DA model: R2X = 0.381, R2Y(cum) = 0.993, Q2(cum) = 0.849) (Fig. 7B). A total of 1452 changed metabolites were identified with the threshold of variable importance in the projection (VIP) ≥ 1 and p ≤ 0.05. In particular 162 differential metabolites were identified between hADMSCs-Exo-treated group and control group, while 267 differential metabolites were identified between hADMSCs-Exo-treated group and the regression group (Fig. 7C). Information of the top 20 most altered metabolites (VIP, p value and fold change) in hADMSCs-Exo-treated group compared to REG or LEG were listed in Additional file 1: Table S4 and Table S5, respectively. As presented in the clustered heatmap (Fig. 7D), Oleamide, sphingosine, FMN, sphinganine, tridemorph ranked by the front of significantly up-regulated metabolites, while the most significantly down-regulated metabolites were betaine, choline, D-Fructose, 2-Hydroxy-2-ethylsuccinic acid.

Through pathway analysis of those differential metabolites, we noticed that lipid and energy metabolic pathways were enriched in comparison between hADMSCs-Exo with REG group or hADMSCs-Exo with LFG group, such as PPAR signaling pathway, choline metabolism, and fatty acid metabolism (Fig. 7E, F). hADMSCs-Exo perturbated metabolomic profiling in fibrotic liver tissue. The increased liver levels of sphingolipid and sphinganine and decreased levels of betaine, glycerophosphocholine (GPC) were reversed in the hADMSCs-Exo-treated group compared with those in the LFG and REG (Fig. 7G). Through ELISA, levels of choline, phosphorylcholine, phosphatidylcholine and sphingomyelin were determined to be reduced in hADMSCs and hADMSCs-Exo groups (p < 0.05) compared to LFG and REG (Fig. 7H). These results suggested that the mechanisms of hADMSCs-Exo against liver fibrosis might involve in the regulation of choline metabolism. The discovery is worthy of further study, regarding the importance of choline metabolism and its regulation of PI3K/AKT signaling.

### hADMSCs-Exo regulates the choline metabolism, which involved in PI3K/AKT/mTOR signaling pathway to anti-liver fibrosis

**Fig. 8:**
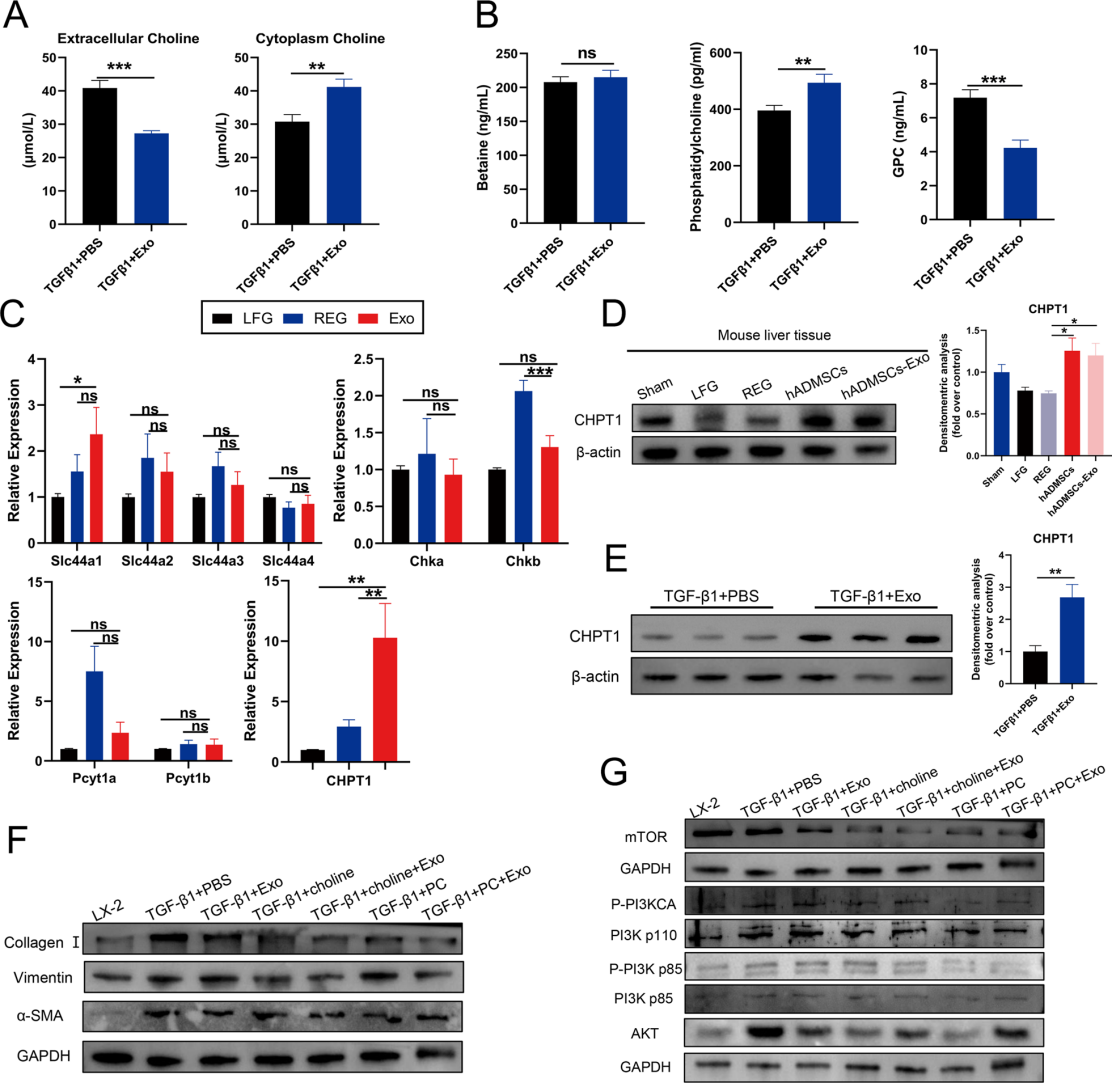
hADMSCs-Exo regulates the choline metabolism, which involved in PI3K/AKT/mTOR signaling pathway to anti-liver fibrosis. **A** Quantitative analyses of extracellular and intracellular choline content in activated LX-2 cells or incubated with hADMSCs-Exo. **B** Quantitative analyses of total intracellular betaine, phosphatidylcholine and glycerophosphocholine content. **C** The mRNA levels of choline metabolism related genes were determined by qRT–PCR in livers of mice. **D**, **E** The protein levels of CHPTI were determined by western blotting in the liver tissues of the mice with different treatments and HSCs incubated with hADMSCs-Exo. **F**, **G** The expression level of profibrogenic markers (Collagen I, Vimentin and α-SMA) and PI3K/AKT signalling pathway proteins were determined by western blotting in activated LX-2 cells incubated with hADMSCs-Exo and treated (or not treated) with choline or phosphorylcholine. GAPDH was used as a loading control. Exo, exosomes. LFG, liver fibrosis group, REG, regression group. *Slc44a1-4,* Solute Carrier Family 44 Member*. Chka,* Choline Kinase Alpha*. Chkb,* Choline Kinase beta*. Pcyt1a,* Phosphate Cytidylyltransferase 1A*. Pcyt1a,* Phosphate Cytidylyltransferase 1B*. CHPT1,* Choline Phosphotransferase 1*.* CHPT1, diacylglycerol cholinephosphotransferase 1. PC, phosphorylcholine. Data expressed as the mean ± SEM (n = 3 independent experiments). ns, not significant, *p < 0.05, **p < 0.01, ***p < 0.001 and ****p < 0.0001

To illustrate the role of hADMSCs-Exo in choline metabolism, we counted changes of intracellular and hepatocyte cytoplasm metabolites as well as expression of metabolic genes following hADMSCs-Exo treatment. Secreted and intracellular levels of choline were detected by performing ELISA on cell supernatants and whole cell lysates respectively. We found that hADMSCs-Exo promoted uptake of the choline by the activated LX-2 cells (Fig. 8A). Meanwhile, intracellular betaine, phosphatidylcholine and GPC levels. The results showed that decreased total intracellular GPC and increased phosphatidylcholine content but no change in betaine content in hADMSCs-Exo-treated cells (Fig. 8B). Next, we examined expression of genes encoding key enzymes in choline metabolism by qRT–PCR. The results revealed that the expression of genes coding for the choline transporter CTL1 (*Slc44a1*, *Slc44a2*, *Slc44a3*, *Slc44a4*), choline kinase alpha (*Chkα*, *Chkβ*) and phosphocholine cytidylyltransferase (*Pcyt1α*, *Pcyt1β*) had no statistically significant differences between hADMSCs-Exo-treated group and LEG or REG. However, exposure to hADMSCs-Exo increased transcription of *Chpt1a*, which encodes the choline phosphotransferase 1 (CHPT1) (Fig. 8C). These results were consistent with the transcriptome results (Additional file 1: Fig. S9). Furthermore, CHPT1 was confirmed to be augmented by hADMSCs-Exo both in vivo (Fig. 8D) and in vitro (Fig. 8E) by western blot analysis, while CHPT1 induction correlated with phosphatidylcholine biosynthesis from CDP-choline, suggesting that hADMSCs-Exo might activate activity of CHPT1 and played a central role in the formation and maintenance of vesicular membranes.

We further investigated whether supplementing choline or phosphorylcholine synergize therapeutic effect of hADMSCs-Exo in LX-2 cells. As shown in Fig. 8F and Additional file 1: Fig. S10A, following supplementation with hADMSCs-Exo and 10 mM choline or 10 mM phosphorylcholine simultaneously in vitro, pro-fibrogenic protein expression of α-SMA, collagen I and vimentin significantly decreased compared to either individually. This demonstrated that the hADMSCs-Exo and choline synergistically enhanced anti-hepatic fibrosis efficacy. To determine whether hADMSCs-Exo-mediated enhanced choline uptake affects PI3K/AKT/mTOR signaling pathway in LX2 cells, we further examined protein expression and activated status of key proteins related to the PI3K/AKT/mTOR signaling pathway by western blot. Compared to PBS control group, addition of choline or phosphorylcholine resulted in decreases in the p-PI3K/PI3K ratio, lower AKT and mTOR levels (Fig. 8G, Additional file 1: Fig. S10B). These data support a role for hADMSCs-Exo in choline-mediated inhibition of the PI3K/AKT/mTOR signaling. In conclusion, these aforementioned data strongly demonstrated that hADMSCs-Exo played a vital role in alleviating LX2 cell activation and suppressing the progression of liver fibrosis through regulating the choline metabolism and inhibiting the PI3K/AKT/mTOR signaling pathway.

## Discussion

Mesenchymal stem cells (MSCs) have shown great capacity for hepatic regeneration and immunomodulation in treatment of liver disorders [19]. However, heterogeneity and immunogenicity of exogenous stem cells may lead to unpleasant effects and potential risk of immune rejection. In this respect, MSCs-derived exosomes (MSCs-Exo) based therapy is evaluated as a promising strategy for patients with liver diseases [20]. MSC-Exo has emerged as a promising candidate for therapeutic application in a variety of liver diseases. Recent studies demonstrated various MSCs-Exo could restore liver homeostasis and enable hepatocytes to recover, repair and regenerate through the transfer of their contents to injured cells, indicating a great therapeutic potential in treatments of liver fibrosis [34, 35]. Growing evidence indicates that hADMSCs-Exo exhibited a safe and effective therapeutic effect in liver fibrosis [25, 35, 36]. In our study, we observed that hADMSCs-Exo were capable to be internalized and integrated into LX-2 cells, regulating cellular phenotypes and inhibiting their proliferation by arresting cell cycle at G1 phase and inducing apoptosis. These observations laid the foundation for the therapeutic effect of hADMSCs-Exo in liver diseases.

Importantly, the therapeutic effect of hADMSCs-Exo appears enhanced over the effect of the parent hADMSCs in terms of reducing hepatocellular injury. This may be because hADMSCs-Exo fuse with, and then reduce hepatocyte apoptosis by direct releasing protective nucleic acids or proteins, such as lncRNA H19 [37]. Previous studies showed that hADMSCs-Exo could reduce NLRP3 inflammasome activation in macrophages and enhance ant-inflammatory responses of natural killer T-cells in liver disease [38, 39]. We found that hADMSCs-Exo significantly decreased inflammatory cytokines to inhibit inflammatory response. This is consistent with reports previously showing that BMSCs- and UCSCs-derived exosomes could promote liver tissue repair by reducing the inflammatory response [40–42]. Therefore, together with our results, these findings further prove that hADMSCs-Exo has the potential as a potent antifibrotic agent for the treatment of liver fibrosis.

Emerging evidence suggested that MSCs-Exo could inhibit liver fibrosis by multiple processes, including autophagy, TGF-β/smad, Wnt/β-catenin, LPS/TLR4, EMT/ERK1, PPARγ, NF-κB pathway [43]. Among them, PI3K/AKT signaling played an essential role in formation and progression of liver fibrosis, which is abnormally activated in fibrotic liver tissue [44]. Previous studies have confirmed that inhibition of PI3K/AKT signaling pathway attenuated ECM synthesis and HSC proliferation to block the progression of hepatic fibrosis [45, 46]. In the present study, we revealed that hADMSCs-Exo meditated PI3K/AKT/mTOR signaling inhibition was essential for suppressing both TGF-β1- induced HSC activation and liver fibrotic ECM deposition. Similarly, another study demonstrated that BMSCs-originated exosomal circDIDO1 suppressed the proliferation, reduced pro-fibrotic markers, and induced apoptosis as well as cell cycle arrest in hepatic stellate cells (HSCs) by blocking PTEN/AKT pathway [43]. These results indicate that AKT pathway is an important signaling pathway associated with exosomes in liver fibrosis. Akt proteins (also known as PKB) are serine-threonine kinases that play a major role in cell growth, cell proliferation and glucose/fat metabolism [47, 48]. In turn, metabolism intersects with a well-described function of PI3K/AKT signaling [49].

Furtherly, we performed metabolomics to illustrate key metabolites associated with repair function of hADMSCs-Exo. Liver plays an important role in maintaining the homeostasis of body metabolism, while the process of liver fibrosis is closely linked to metabolism. Conversely, improvements in metabolic reprogramming could facilitate reversal of liver fibrosis. Exosomes act as regulators of metabolism, while those altered metabolites have a major role in blocking HSC activation and fibrogenesis [50, 51]. Choline, an essential nutrient, is a constituent of cell and mitochondrial membranes and of the neurotransmitter acetylcholine [52]. Clinical and experimental evidence has shown that low dietary choline perturbs mitochondrial bioenergetics [53] and fatty acid beta-oxidation [54], resulting in fatty liver and liver damage, which in turn progresses to steatohepatitis, fibrosis, cirrhosis, and liver cancer [52, 55, 56]. A significantly increased level of phosphocholine in liver tissue also indicates a disruption in membrane fluidity due to lipid peroxidation, resulting in membrane damage and liver injury [57, 58]. The previous reports also showed that CCl_4_ exposure easily causes the disorder of PEs and PCs metabolism and can strongly induce degradation of lipid peroxides and oxidative damage due to excessive free radicals (ROS), resulting in the destruction of cell membrane structure [59, 60]. In the present study, hADMSCs-Exo could promote choline absorption. The newly taken-up choline did not account for free choline accumulation, but was rapidly converted to phosphatidylcholine by upregulated CHPT1 via the Kennedy pathway. The level of phosphocholine decreased significantly in hADMSCs and hADMSCs-Exo treatment groups, suggesting that hADMSCs-Exo regulated phospholipid metabolism and was essential for the maintenance of normal function and properties of cell membrane, including its fluidity and permeability. Moreover, choline supplementation cooperated with hADMSCs-Exo in anti-hepatic fibrosis and inhibited PI3K/AKT/mTOR pathway. Therefore, supplementation of choline is helpful to normalize impaired cholesterol metabolism, which is supposed to protect against liver damage and prevent deterioration of fibrosis sufficiently [61]. Moreover, our finding suggested that hADMSCs-Exo could lessen accumulation of lipids and restore the abnormal choline metabolism, which contributed to alleviating hepatocyte damage and fibrosis. On this basis, choline supplementation is supposed to exert a synergistic effect with hADMSCs-Exo in anti-hepatic fibrosis. Moreover, we suggest that the clinical efficacy of antifibrotic drugs related to lipid metabolites (polyene phosphatidylcholine, PPAR synthetic ligand thiazolidinediones) and whether lipid metabolites can be used as biomarkers for predicting the severity of hepatic fibrosis should be further explored.

Recently, exosome-mediated intercellular transfer of different nucleic acids and exosomal proteins has been found implicated in fine-tuning of liver fibrosis [34, 62, 63]. It is important to note that most of the current studies mainly focus on miRNAs carried by exosomes, such as miR-181-5p and miR-122 [36, 64], which were shown to contribute to intercellular communications mediating fibrogenic signaling [36, 64]. Another report showed that miRNAs are not the only players behind exosomes, rather, components including cytokines and proteins, lipids and others carried by exosomes might perform substantial role to motivate exosomes for their terminal accomplishment [65]. Therefore, crucial effective contents of hADMSCs-Exo (including miRNAs, mRNA, lncRNA, DNA, proteins, and lipid) and specific molecular mechanisms on antifibrotic function require further detailed explorations through omics technology. Moreover, some other limitations should be acknowledged in our study. A long-term pharmacological study and pertinent validation experiments in large animals should be conducted before clinical application of hADMSCs-Exo. In addition, considering that exosomes consumption in veins and it is still not feasible to perform biweekly large quantities of hADMSCs-Exo in patients with hepatocirrhosis by intravenously injection, further surface modification of hADMSCs-Exo targeting on damaged hepatocyte and large-scale exosome production should be addressed in the future research.

In conclusion, our study identified the ability of exosomes isolated from hADMSCs in ameliorating liver fibrosis progression in a dose-dependent manner. Improvements in choline-phosphatidylcholine metabolism and inhibition of PI3K/AKT/mTOR signaling appeared to be the underlying mechanism that restored cell membrane, attenuated stellate cell activation and suppressed the progression of liver fibrosis. Our findings provide important insights into the molecular mechanisms underlying the antifibrotic effects of hADMSCs-Exo with a focus on metabolic homeostasis, and help inform the development of a safe and effective therapeutic. The current study is, therefore, an important step before the clinical usage of hADMSCs-Exo for chronic liver fibrosis.

## Supplementary Information

**Additional file 1: Figure S1.** Characterization of hADMSCs. **Figure S2. **TGF-β1 activates hepatic stellate cells leading to ECM deposition and EMT progression.** Figure S3.** Inhibition of activated HSCs proliferation and activation by hADMSCs.** Figure S4.** Liver cirrhosis was successfully induced in C57BL/6J mice with CCl4 i.p. injections twice a week for 8 weeks.** Figure S5. **Ex vivo organ distribution of PKH26-hADMSCs-Exo in CCl4-induced liver fibrosis mouse model.** Figure S6. **Liver stiffness of mouse with different treatments was measured by shear wave elastrography (SWE) at predetermined time. **Figure S7. **Representative histopathology analysis of liver sections after 0, 2 and 4 weeks of hADMSCs-Exo treatment. **Figure S8.** GO and KEGG pathway analyses between LFG vs hADMSCs-Exo or REG. **Figure S9. **Western blot assay for AKT, mTOR and phosphorylated and total PI3K p85 and PI3K p110 in vivo (A) and in vitro (B).** Figure S10. **The gene coding for the key metabolite expression analysis in transcriptome. **Figure S11. **Western blot assay for pro-fibrogenic and PI3K/AKT/mTOR signaling protein expression in aHSCs following supplementation with hADMSCs-Exo and 10 mM choline or 10 mM phosphorylcholine simultaneously in vitro. **Table S1.** Primer sequences used in reverse transcription quantitative PCR (RT-qPCR). **Table S2.** Antibodies utilized in Western blot, immunohistochemistry or Immunofluorescence staining. **Table S3.** The top 25 significant KEGG pathway between hADMSCs-Exo group (n=3) and regression group (REG, n=3). **Table S4.** Significant metabolites between hADMSCs-Exo treatment group (n=6) and regression group (n=6) in the liver tissue samples. **Table S5.** Significant metabolites between hADMSCs-Exo treatment group (n=6) and liver fibrosis group (n=6) in the liver tissue samples.

## Data Availability

Raw RNA-seq data was deposited in Gene Expression Omnibus (GEO) (http://www.ncbi.nlm.nih.gov/geo/) database with the data set identifier GSE199732.

## Abbreviations

hADMSCs-Exo: Exosomes derived from human adipose mesenchymal stem cells
HSCs: Hepatic stellate cells
MSCs: Mesenchymal stem cells
EV: Extracellular vesicle
TGF-β1: Transforming growth factor-β1
SWE: Shear wave elastrography
LFG: Liver fibrosis group
REG: Regression group
Choe: Extracellular free choline
Choi: Intracellular free choline
CHK: Choline kinase
PCho: Phosphocholine
CCT, CTP: Phosphocholine cytidylyltransferase
CDP-Cho: Cytidine diphosphate-choline
CHPT1: Diacylglycerol cholinephosphotransferase 1
PtdCho: Phosphatidylcholine
GPC: Glycerophosphocholine
ECM: Extracellular matrix
EMT: Epithelial-to-mesenchymal transition
