# Supplementary material for: Exosomes derived from human adipose mesenchymal stem cells ameliorate hepatic fibrosis by inhibiting PI3K/Akt/mTOR pathway and remodeling choline metabolism
Source: J Nanobiotechnology. 2023 Jan 25;21:29. doi: 10.1186/s12951-023-01788-4 (PMC9878808; doi:10.1186/s12951-023-01788-4)
Supplement: Supplementary file 1 — Additional file 1: Figure S1. Characterization of hADMSCs. Figure S2. TGF-β1 activates hepatic stellate cells leading to ECM deposition and EMT progression. Figure S3. Inhibition of activated HSCs proliferation and activation by hADMSCs. Figure S4. Liver cirrhosis was successfully induced in C57BL/6J mice with CCl4 i.p. injections twice a week for 8 weeks. Figure S5. Ex vivo organ distribution of PKH26-hADMSCs-Exo in CCl4-induced liver fibrosis mouse model. Figure S6. Liver stiffness of mouse with different treatments was measured by shear wave elastrography (SWE) at predetermined time. Figure S7. Representative histopathology analysis of liver sections after 0, 2 and 4 weeks of hADMSCs-Exo treatment. Figure S8. GO and KEGG pathway analyses between LFG vs hADMSCs-Exo or REG. Figure S9. Western blot assay for AKT, mTOR and phosphorylated and total PI3K p85 and PI3K p110 in vivo (A) and in vitro (B). Figure S10. The gene coding for the key metabolite expression analysis in transcriptome. Figure S11. Western blot assay for pro-fibrogenic and PI3K/AKT/mTOR signaling protein expression in aHSCs following supplementation with hADMSCs-Exo and 10 mM choline or 10 mM phosphorylcholine simultaneously in vitro. Table S1. Primer sequences used in reverse transcription quantitative PCR (RT-qPCR). Table S2. Antibodies utilized in Western blot, immunohistochemistry or Immunofluorescence staining. Table S3. The top 25 significant KEGG pathway between hADMSCs-Exo group (n=3) and regression group (REG, n=3). Table S4. Significant metabolites between hADMSCs-Exo treatment group (n=6) and regression group (n=6) in the liver tissue samples. Table S5. Significant metabolites between hADMSCs-Exo treatment group (n=6) and liver fibrosis group (n=6) in the liver tissue samples. [file 12951_2023_1788_MOESM1_ESM.docx]

**Additional files for**

**Exosomes Derived from Human** **Adipose Mesenchymal Stem Cells** **Ameliorate Hepatic Fibrosis by Regulating Choline Metabolism and PI3K/Akt/mTOR Pathway**

Zilong Zhang^1^†, Jin Shang^1^†, Qinyan Yang^1^†, Zonglin Dai^1^, Yuxin Liang^1^, Chunyou Lai^1^, Tianhang Feng^1^, Deyuan Zhong^1^, Haibo Zou^1^, Lelin Sun^1^, Yuhao Su^1^, Su Yan^1^, Jie Chen^2^, Yutong Yao^1^, Ying Shi^1^*, Xiaolun Huang^1^*

1. School of Medicine, University of Electronic Science and Technology of China, Chengdu 610054, Sichuan, China.
2. Department of Core laboratory, Sichuan Provincial People's Hospital, University of Electronic Science and Technology of China, Chengdu, China.

**†These authors have contributed equally to this work.**

***Corresponding author:**

Xiaolun Huang: University of Electronic Science and Technology of China. No. 32, West Second Section, First Ring Road, Qingyang District, Chengdu 610072, Sichuan, China.

Email: huangxiaolun@med.uestc.edu.cn

Tel: +86-87393753

Fax: 86- 28 85423052

Ying Shi: School of Medicine, University of Electronic Science and Technology of China, Chengdu 610054, Sichuan, China.

Email: shiying_uestc@uestc.edu.cn.

**Figure S1** Characterization of hADMSCs. **Figure S2** TGF-β1 activates hepatic stellate cells leading to ECM deposition and EMT progression. **Figure S3** Inhibition of activated HSCs proliferation and activation by hADMSCs. **Figure S4** Liver cirrhosis was successfully induced in C57BL/6J mice with CCl4 i.p. injections twice a week for 8 weeks. **Figure S5** Ex vivo organ distribution of PKH26-hADMSCs-Exo in CCl4-induced liver fibrosis mouse model. **Figure S6** liver stiffness of mouse with different treatments was measured by shear wave elastrography (SWE) at predetermined time. **Figure S7** Representative histopathology analysis of liver sections after 0, 2 and 4 weeks of hADMSCs-Exo treatment. **Figure S8** GO and KEGG pathway analyses between LFG vs hADMSCs-Exo or REG. **Figure S9** Western blot assay for AKT, mTOR and phosphorylated and total PI3K p85 and PI3K p110 in vivo (A) and in vitro (B). **Figure S10** The gene coding for the key metabolite expression analysis in transcriptome. **Figure S11** Western blot assay for pro-fibrogenic and PI3K/AKT/mTOR signaling protein expression in aHSCs following supplementation with hADMSCs-Exo and 10 mM choline or 10 mM phosphorylcholine simultaneously in vitro.

**Table S1** Primer sequences used in reverse transcription quantitative PCR (RT-qPCR). **Table S2** Antibodies utilized in Western blot, immunohistochemistry or Immunofluorescence staining. **Table S3** The top 25 significant KEGG pathway between hADMSCs-Exo group (n=3) and regression group (REG, n=3). **Table S4** Significant metabolites between hADMSCs-Exo treatment group (n=6) and regression group (n=6) in the liver tissue samples. **Table S5** Significant metabolites between hADMSCs-Exo treatment group (n=6) and liver fibrosis group (n=6) in the liver tissue samples.

**Figure. S1**


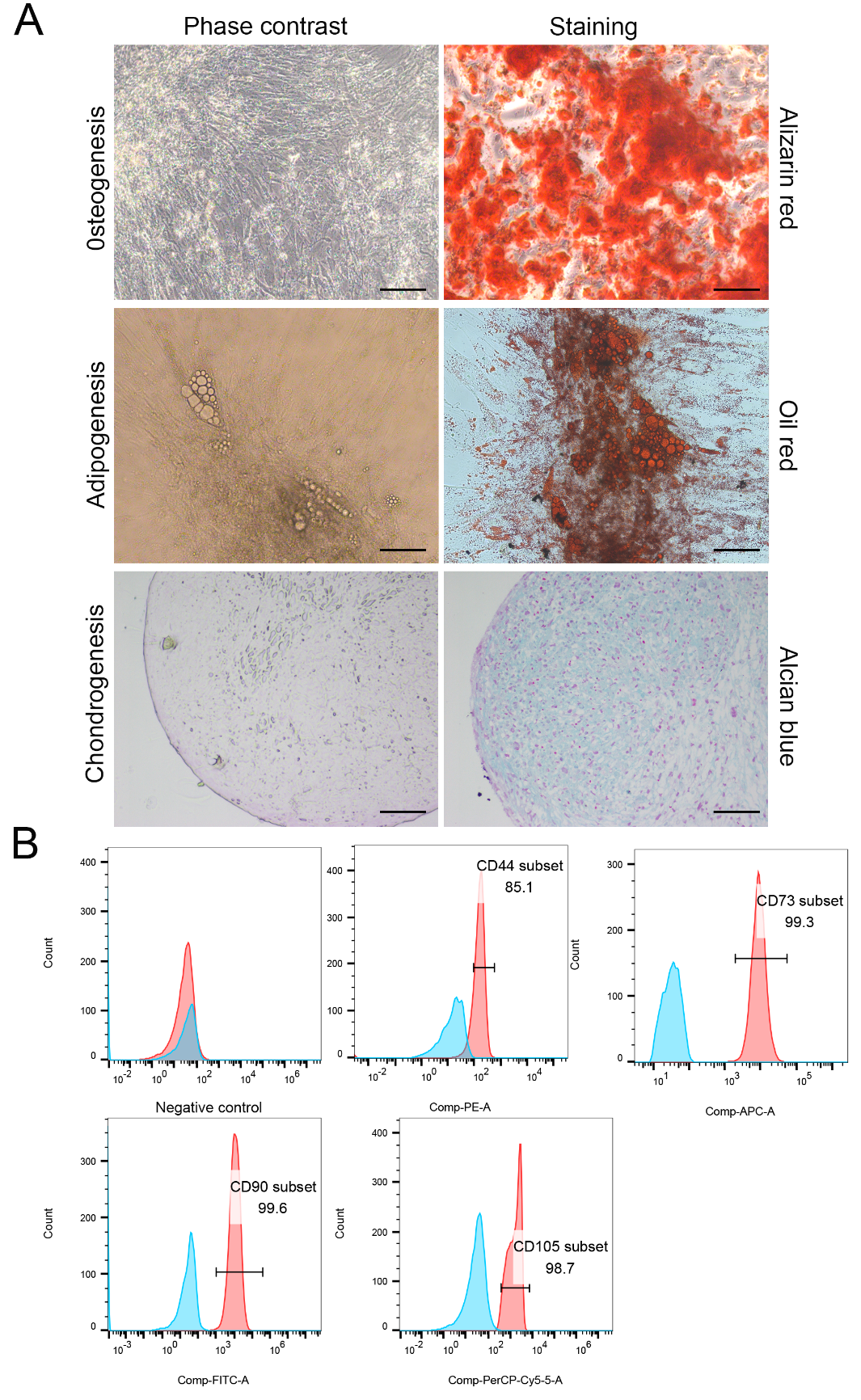


**Figure S1 Characterization of hADMSCs. (A)** The potential of hADMSCs on differentiation of osteoblasts, adipocytes, and chondrocytes in the specified medium was identified by the Alizarin Red staining, Oil Red O staining, and Alcian Blue staining, respectively; Scale bar: 500 μm. **(B)** Flow cytometry detection of the typical surface markers CD44 CD73, CD90, and CD105 in hADMSCs; Blue curves: the isotype controls; red curves: the test samples.

**Figure. S2**


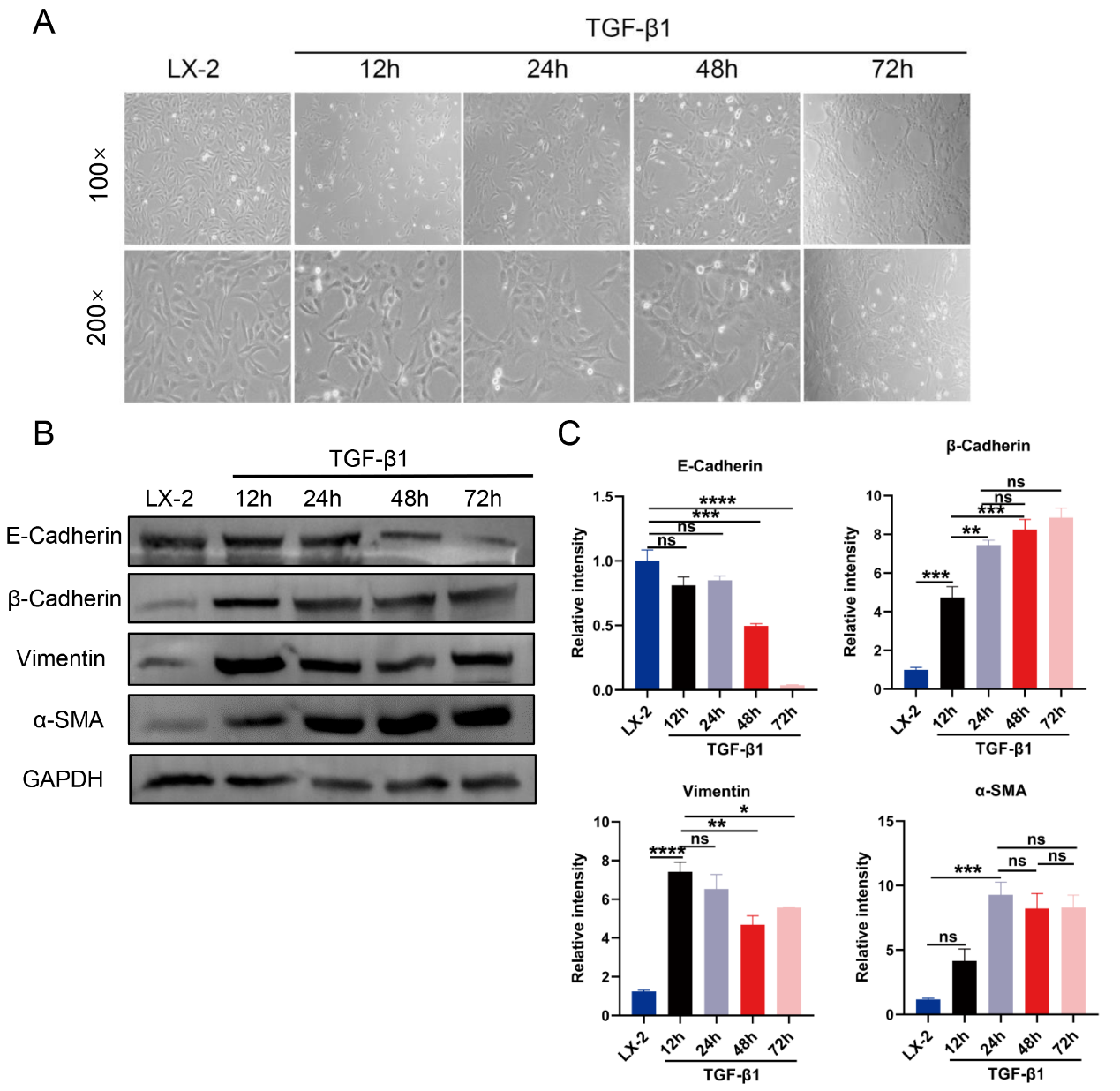


**Figure S2 TGF-β1 activates hepatic stellate cells leading to ECM deposition and EMT progression. (A)** Representative phase-contrast images of quiescent HSCs or treated with 10 ng/ml TGF-β1 for 12h, 24h, 48h and 72h. Original magnifcation, 100× (upper), 200×(lower). **(B)** The protein level of ECM-related marker (α-SMA) and EMT--related marker (Vimentin, β-catenin and E-catenin) were determined by western blot. **(C)** The relative expression levels of α-SMA, Vimentin, β-catenin and E-catenin. Data are presented as means with SEM (n=3 independent experiments). ns, not significant, *p < 0.05, **p < 0.01, ***p < 0.001 and ****p < 0.0001.

**Figure. S3**


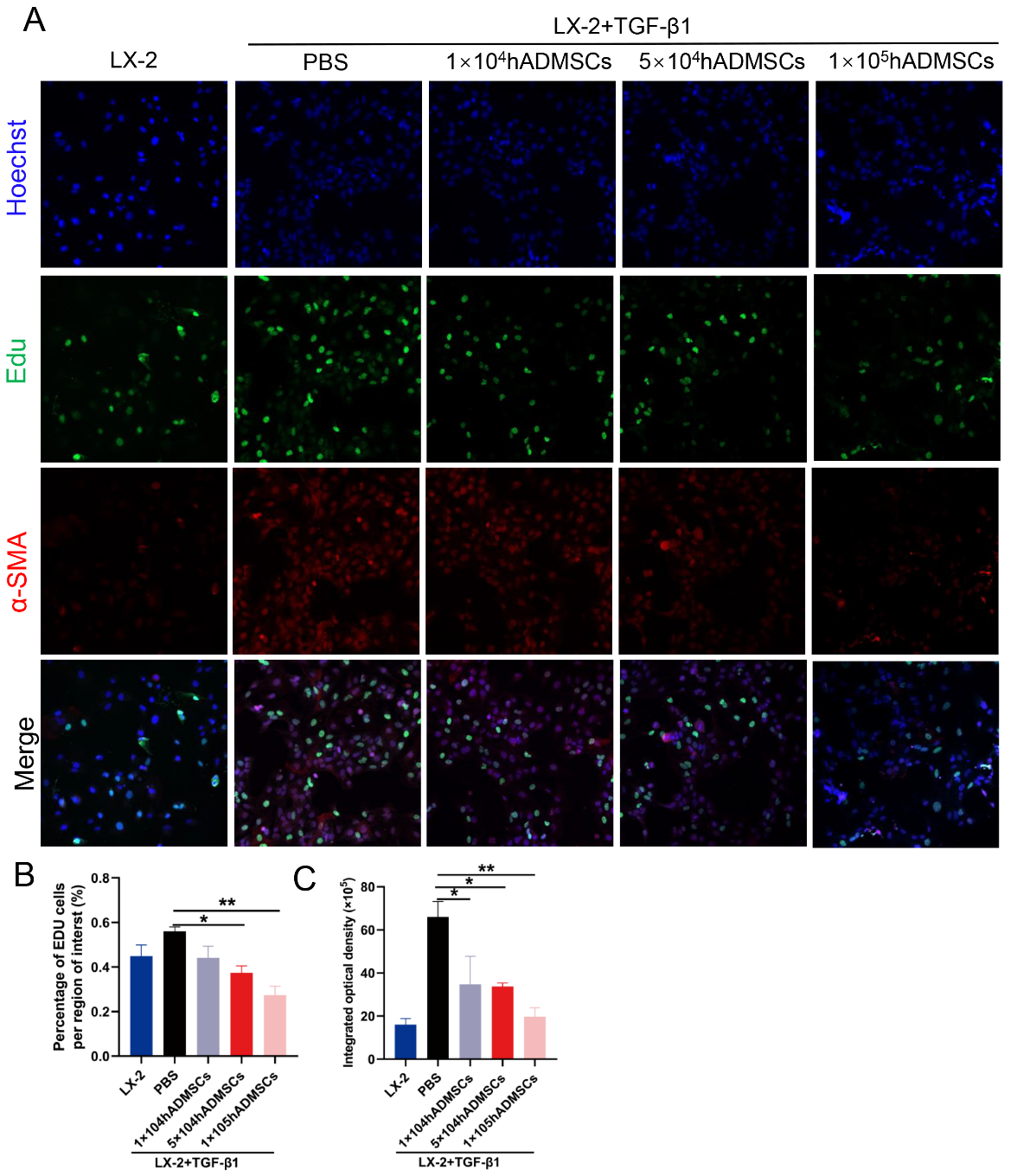


**Figure S3** **Inhibition of activated HSCs proliferation and activation by hADMSCs. (A)** Representative immunofluorescence images of LX-2 cells or co-cultured with hADMSCs at the indicated concentration in the presence of TGF-β1 (10 ng/ml) for 24 h. Scale bars, 100 mm. **(B)** Edu assay showed that hADMSCs could inhibit proliferation of aHSCs. **(C)** α-SMA protein levels in LX-2 or treated with pbs, hADMSCs in the presence of TGF-β1 (10 ng/ml) for 24 h were measured by immunofluorescence staining. Data are presented as means with SEM (n=3 independent experiments). *p < 0.05 and **p < 0.01.

**Figure S4**


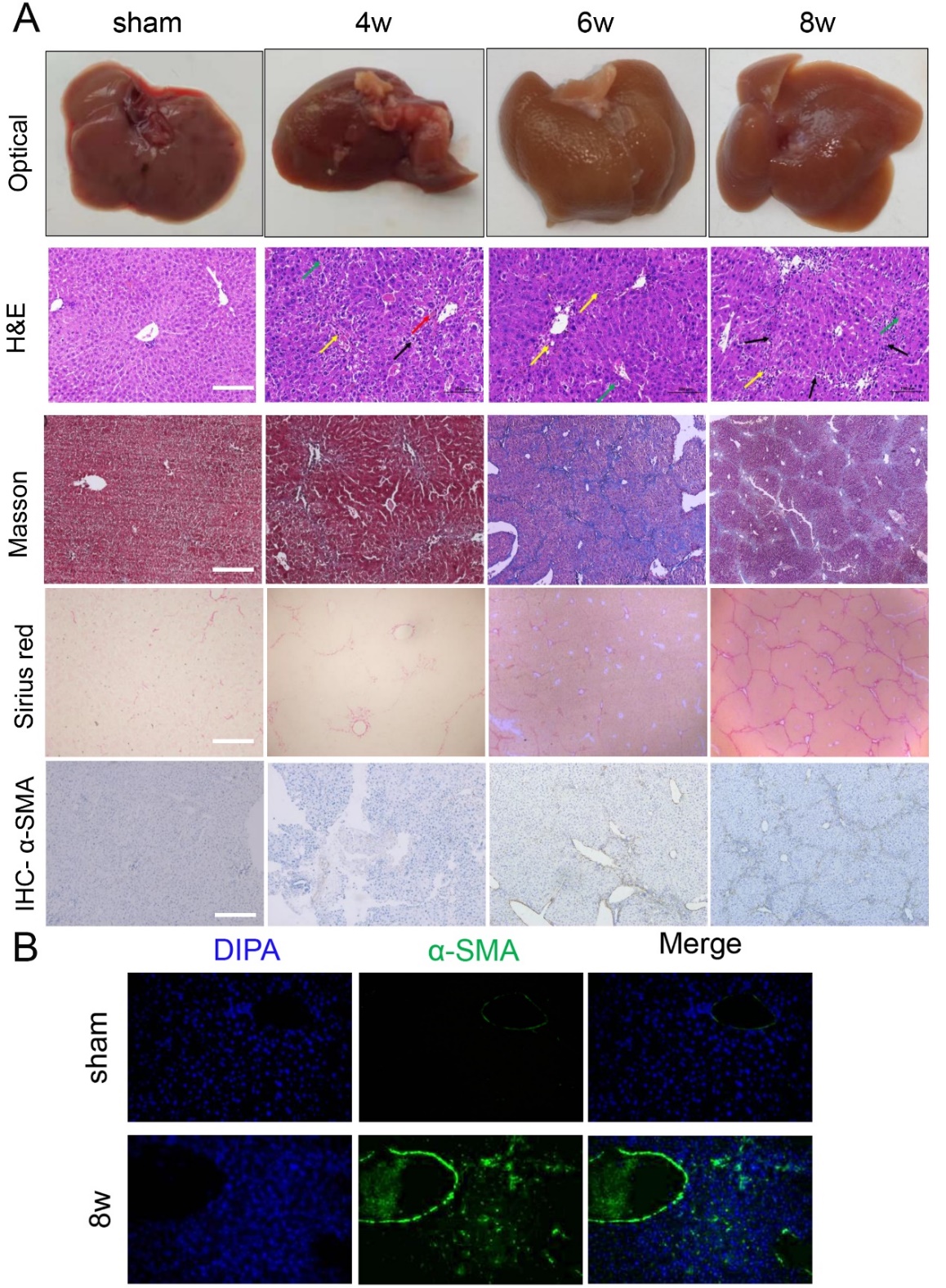


**Figure S4** **Liver cirrhosis was successfully induced in C57BL/6J mice with CCl4 i.p. injections twice a week for 8 weeks.** **(A)** Representative photographs, histopathology and immunohistochemistry of normal and CCl4-induced livers from mice for 4 weeks, 6 weeks and 8 weeks. **(B)** Representative Images of immunohistochemistry staining of α-SMA in normal and CCl4-induced livers from mice for 8 weeks.

**Figure. S5**


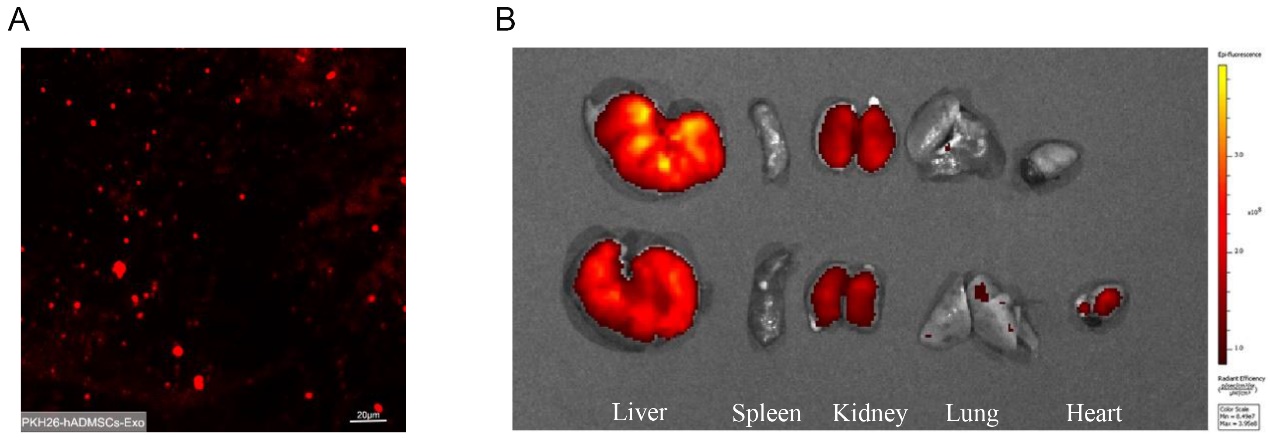


**Figure S5 Ex vivo organ distribution of PKH26-hADMSCs-Exo in CCl4-induced liver fibrosis mouse model. (A)** CLSM images of hADMSCs-Exo labeled with PKH26. **(B)** Ex vivo organ imaging of PKH26-hADMSCs-Exo after 3 days at vital organs, including liver, spleen, kidney, lung and heart.

**Figure. S6**


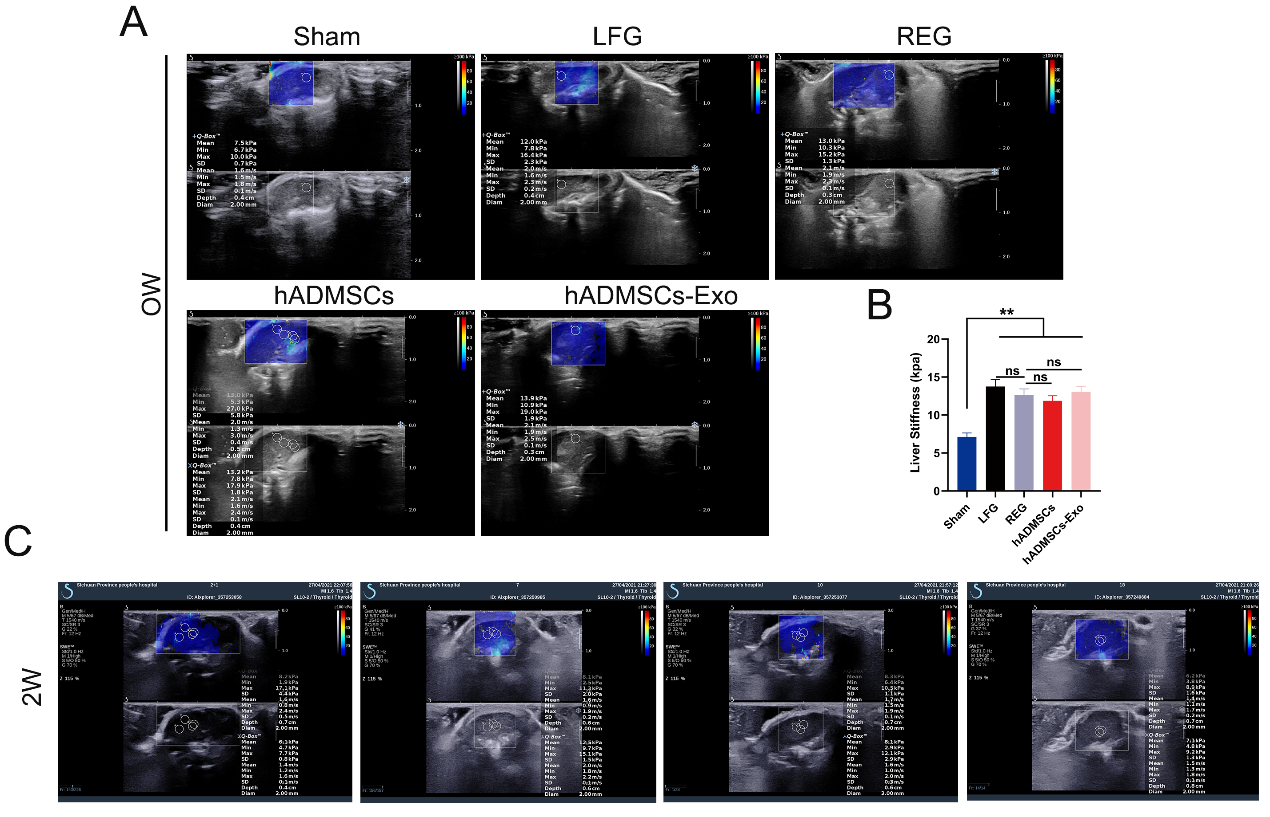


**Figure S6 liver stiffness of mouse with different treatments was measured by** **shear wave elastrography (SWE) at predetermined time. (A)** liver stiffness of mouse with different treatments prior to the start of treatment (baseline). **(B)** SWE verified the successful of modeling**. (C)** liver stiffness of mouse with different treatments during treatment (weeks 2). LFG, liver fibrosis group, REG, regression group. ns, not significant, *p < 0.05, **p < 0.01.

**Figure. S7**


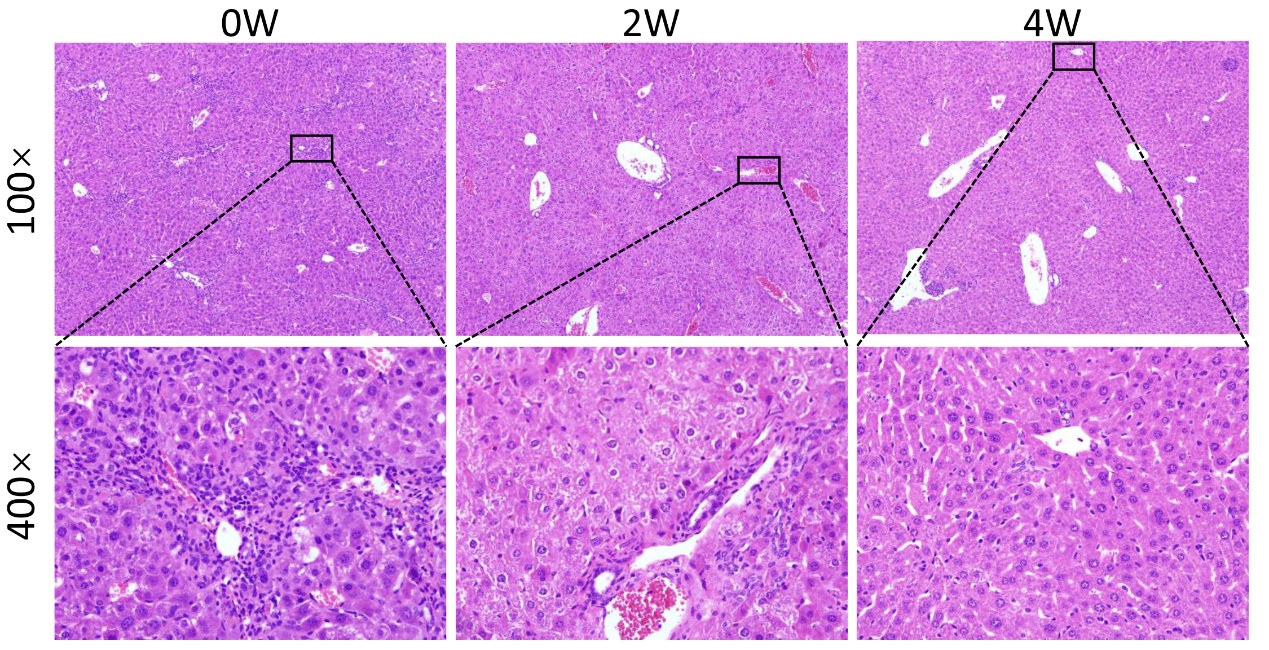


**Figure S7 Representative histopathology analysis of liver sections after 0, 2 and 4 weeks of hADMSCs-Exo treatment.** There are numerous of hepatocytes swelling, cytoplasm loose light dye, intravenous surrounding connective tissue hyperplasia, occasional portal area with a small amount of inflammatory cell infiltration, hepatocyte dotted necrosis, nucleus dissolved in the liver at 2 weeks of hADMSCs-Exo treatment. The results showed that the hepatic fibrosis was not completely resolved after 2 weeks of hADMSCs-Exo treatment.

**Figure. S8**


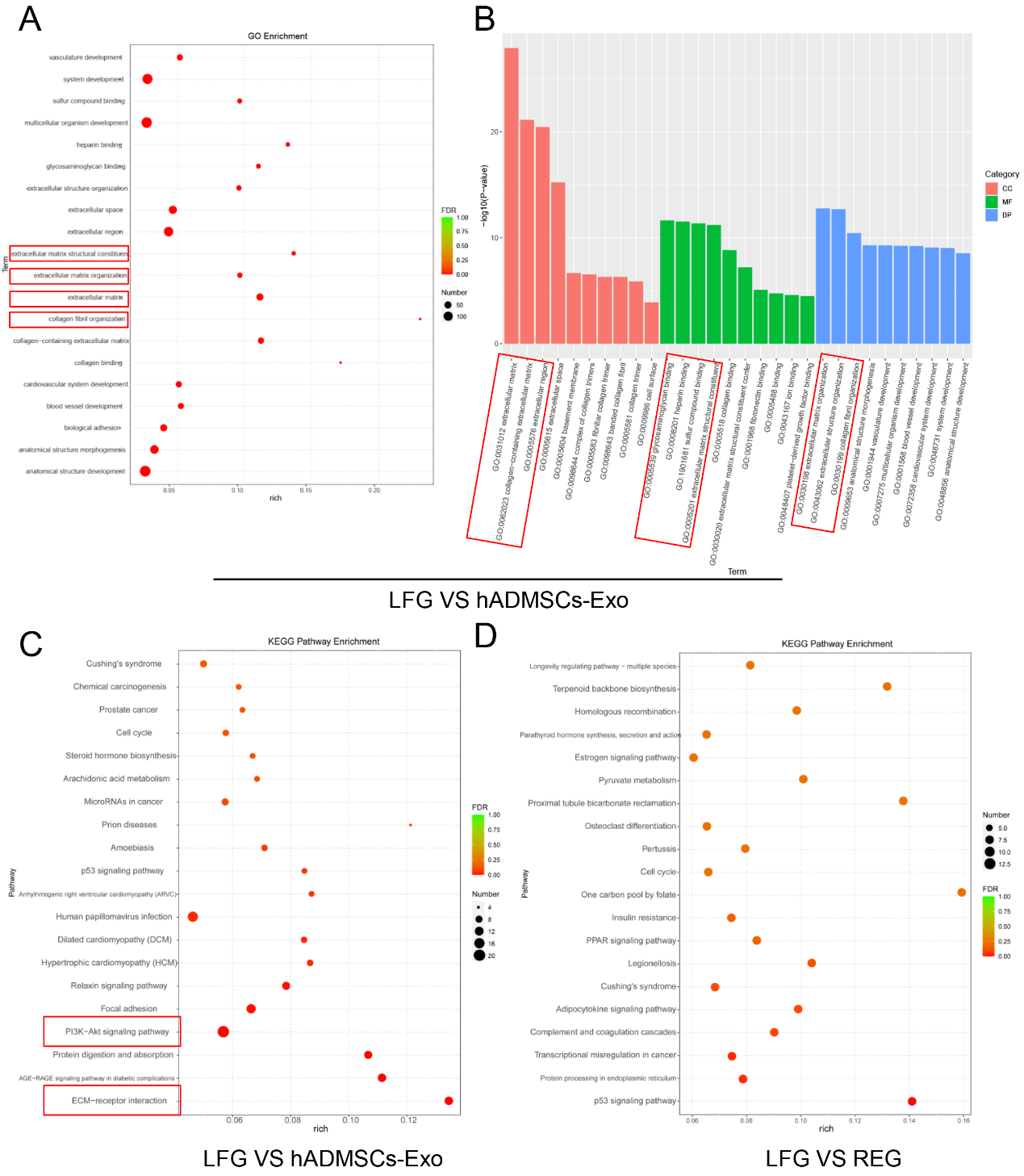
**Figure S8 GO and KEGG pathway analyses between LFG vs hADMSCs-Exo or REG. (A-B)** GO pathway analyses between LFG vs hADMSCs-Exo showed that hADMSCs-Exo might involve in ECM, ECM structural construction, collagen fibril organization. **(C)** KEGG pathway analyses between LFG vs hADMSCs-Exo showed that hADMSCs-Exo involved in ECM-receptor interaction and PI3K/AKT signaling pathway. **(D)** KEGG pathway analyses between LFG vs REG. LFG, liver fibrosis group, REG, regression group, ECM, extracellular matrix.

**Figure. S9**


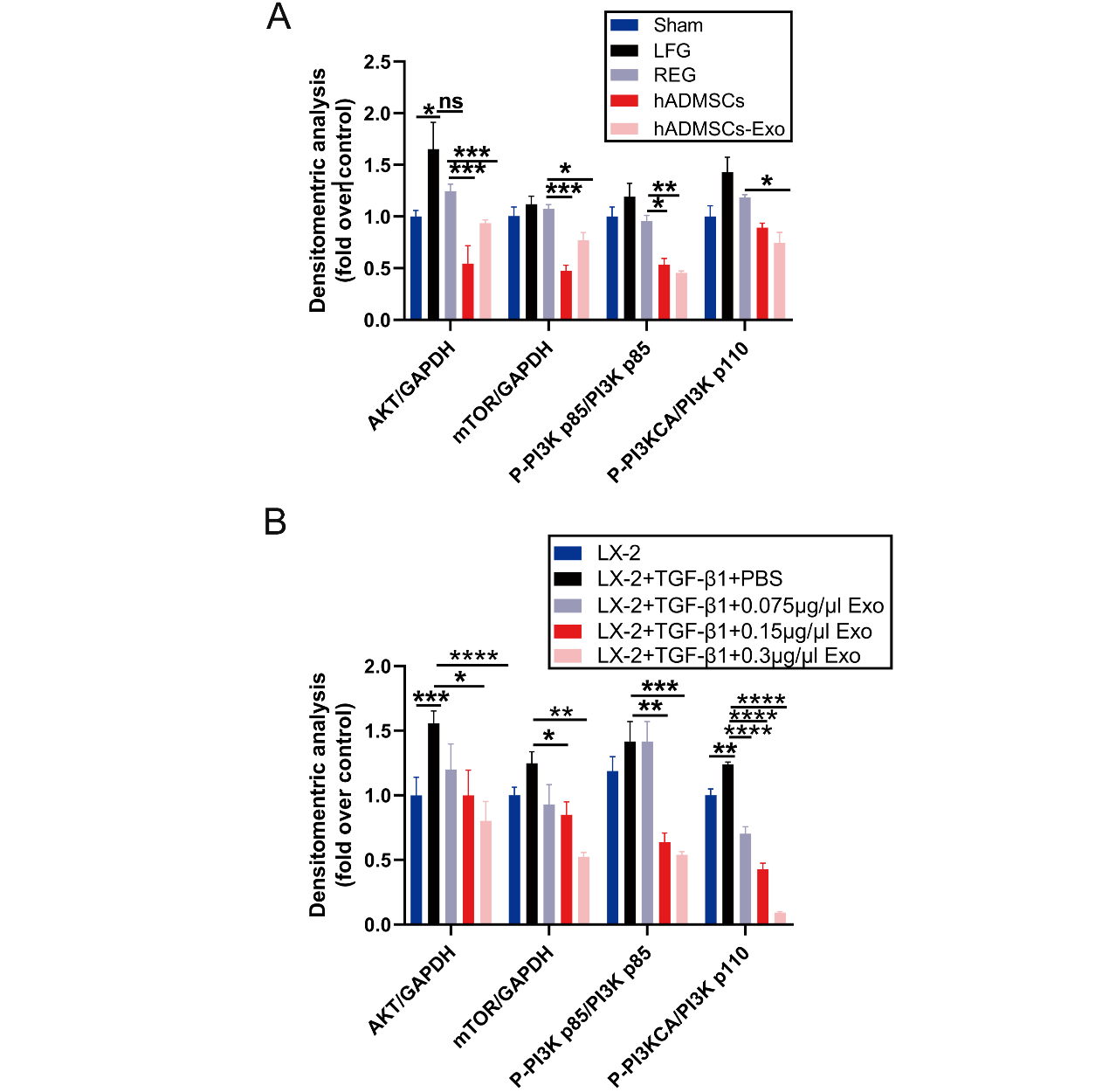


**Figure S9** **Western blot assay for AKT, mTOR and phosphorylated and total PI3K p85 and PI3K p110 in vivo (A) and in vitro (B).** LFG, liver fibrosis group, REG, regression group. Data are presented as means with SEM (n=3 independent experiments). ns, not significant, *p < 0.05, **p < 0.01, ***p < 0.001 and ****p < 0.0001.

**Figure. S10**


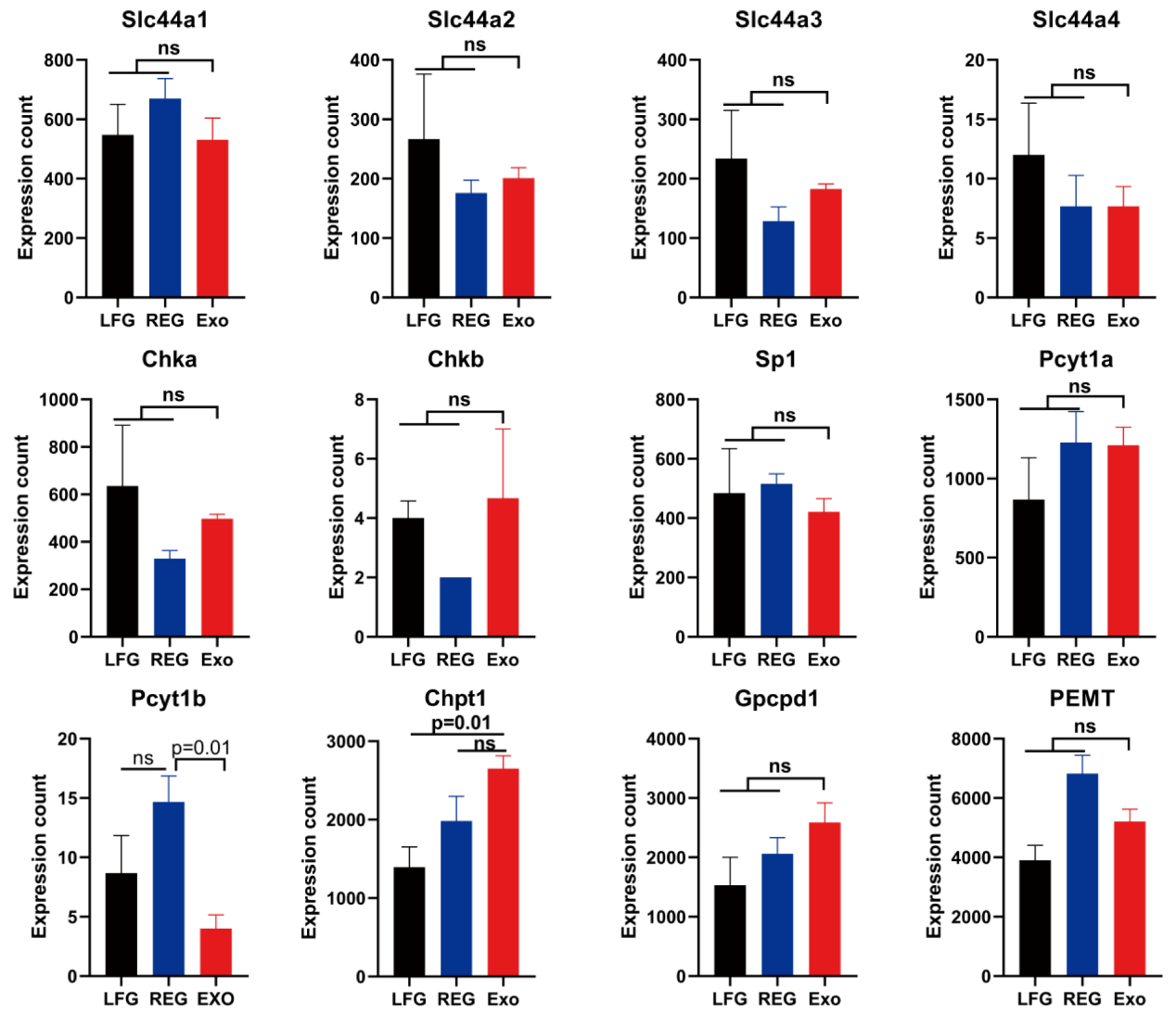


**Figure S10 The gene coding for the key metabolite expression analysis in transcriptome.** *Slc44a1-4,* Solute Carrier Family 44 Member*. Chka,* Choline Kinase Alpha*. Chkb,* Choline Kinase beta*. Sp1,* Sp1 Transcription Factor*. Pcyt1a,* Phosphate Cytidylyltransferase 1A*. Pcyt1a,* Phosphate Cytidylyltransferase 1B*. Chpt1,* Choline Phosphotransferase 1*. Gpcpd1,* Glycerophosphocholine Phosphodiesterase 1. *PEMT,* Phosphatidylethanolamine N-Methyltransferase. Data are presented as means with SEM (n=3). ns, not significant.

**Figure. S11**


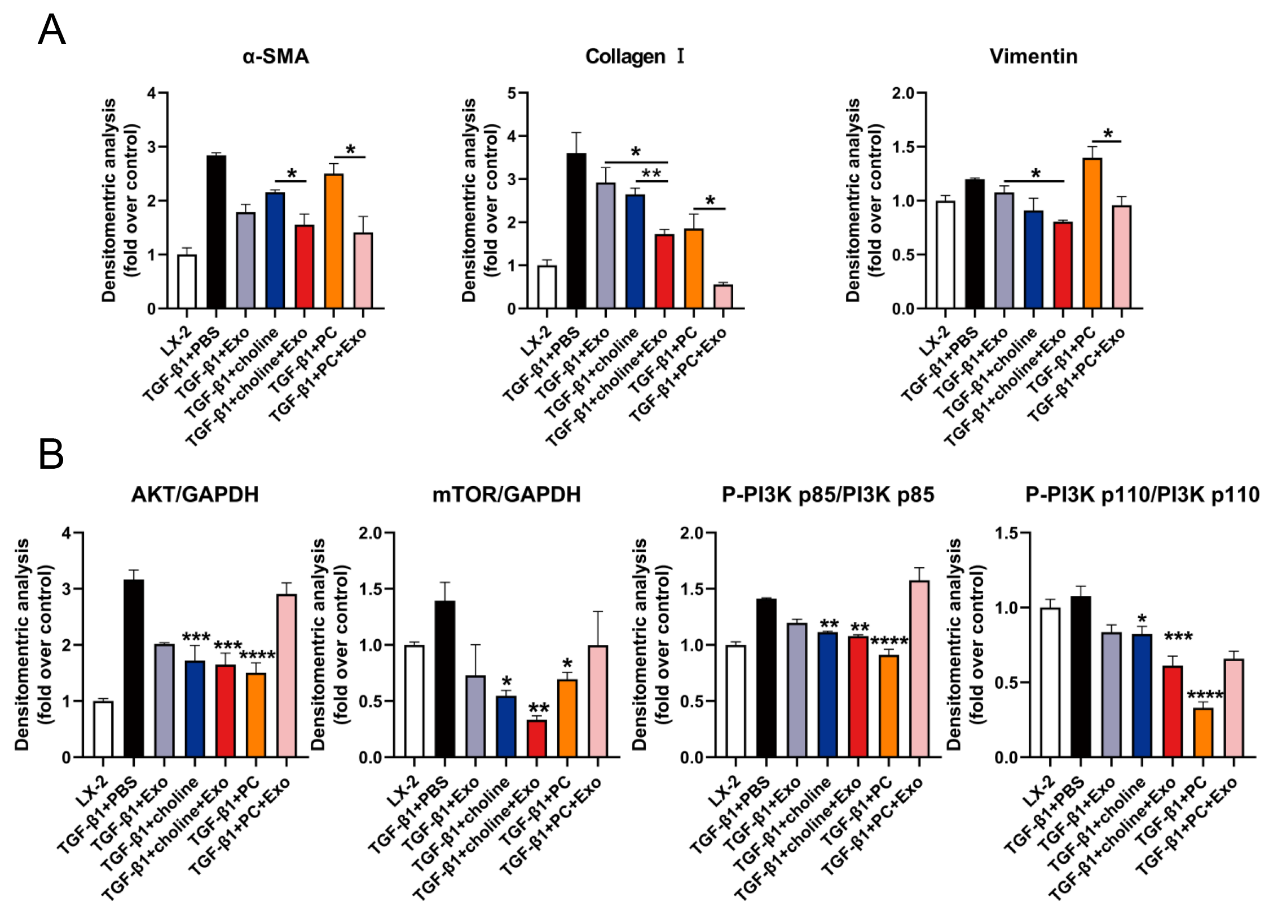


**Figure S11 Western blot assay for pro-fibrogenic and PI3K/AKT/mTOR signaling protein expression in aHSCs following supplementation with hADMSCs-Exo and 10****mM** **choline or 10 mM** **phosphorylcholine simultaneously in vitro**. **(A)** Western blot assay for α-SMA， collagen Ⅰ, vimentin protein expression. **(B)** Western blot assay for AKT, mTOR and phosphorylated and total PI3K p85 and PI3K p110 protein expression, vs. the TGF-β1+PBS group. PC, phosphorylcholine. Data are presented as means with SEM (n=3 independent experiments). ns, not significant, *p < 0.05, **p < 0.01, ***p < 0.001 and ****p < 0.0001.

**Supporting Tables**

Table S1 Primer sequences used in reverse transcription quantitative PCR (RT-qPCR)

| Gene | Forward primer (5′–3′) | Reverse primer (5′–3′) |
| --- | --- | --- |
| Cdkn1a | CCTGGTGATGTCCGACCTG | CCATGAGCGCATCGCAATC |
| Col6a1 | CTGCTGCTACAAGCCTGCT | CCCCATAAGGTTTCAGCCTCA |
| Col6a2 | GCTCCTGATTGGGGGACTCT | CCAACACGAAATACACGTTGAC |
| Lama4 | ATGAGCTGCAAGGAAAACTATCC | CTGTTTCGTTGGCTTCACTGA |
| Itga8 | CGAAGCCGAACTCTTTGTTATCA | GGCCTCAGTCCCTTGTTGT |
| Lamb1 | AGACTTTGGGGGTTCATGTCA | ATCGTCCCGTCTCCTTGTCA |
| MMP2 | GTATTTGATGGCATCGCTCA | CATTCCCTGCAAAGAACACA |
| Tnc | GGTGCGCTACACTTCTGTC | CCTGATACTCCACACCTGGTC |
| Thbs2 | CACGGGAGACAAACTCAGCC | CTGGCAGGGACTTGATAAAATGT |
| Col1a1 | GCCCAGAGATAGAGTGACCTG | CGCACTTCTCGAAAGTTGCTG |
| Col1a2 | GCTCCTGATTGGGGGACTCT | CCAACACGAAATACACGTTGAC |
| IL-10 | CCCATTCCTCGTCACGATCTC | TCAGACTGGTTTGGGATAGGTTT |
| IL-6 | AAGAGCCGGAAATCCACGAAA | GTCTCAAAAGGGTCAGGGTACT |
| TNF-α | CTTTCGGTTGCTCTTTGGTTG | CGACAGCACAAGTCACAGC |
| IL-2 | TGAGCAGGATGGAGAATTACAGG | GTCCAAGTTCATCTTCTAGGCAC |
| IL-1β | AGTTGACGGACCCCAAAAG | AGCTGGATGCTCTCATCAGG |
| AKT | ATGAACGACGTAGCCATTGTG | TTGTAGCCAATAAAGGTGCCAT |
| mTOR | ACCGGCACACATTTGAAGAAG | CTCGTTGAGGATCAGCAAGG |
| Slc44a1 | TTTGCCCAAGCTACCAG | GAGCACAGCGATGGAAGAA |
| Slc44a2 | CCTGGTGCTTGGCTATGG | CAAGGTCCAGGGAGA |
| Slc44a3 | GGTCATTTTGGGATTGCTGT | ACTGAGGTCGTTGGTGTAGTCA |
| Slc44a4 | ACTCTGTCCCCGTTTCCTTC | AAGTTGATGTTGGGGAGTGG |
| Chka | GGGTGGTCTCAGTAACATGCT | GAACCCTGGACTCACCATCTT |
| Chkb | AGGATGCTAAGTGCCCAGAG | TCACGGGACAAACGCTCAG |
| Pcyt1a | GATGCACAGAGTTCAGCTAAAGT | TGGCTGCCGTAAACCAACTG |
| Pcyt1b | GAAACAGGTATCCCAAAATCCCT | GCATGTGTGCTCTATTTCCTCCA |
| CHPT1 | ACTGAGATCCAGGTAGCTTTAGT | GTAGACCCATTCTTGCCAACA |

Table S2 Antibodies utilized in Western blot, immunohistochemistry or Immunofluorescence staining

| Antibodies | SOURCE | IDENTIFIER |
| --- | --- | --- |
| GAPDH (WB:1/10000) | Proteintech | Cat No: 10494-1-AP |
| β-actin (WB:1/10000) | Proteintech | Cat No: 66009-1-Ig |
| Anti-rabbit IgG, HRP-linked Antibody (WB:1/5000) | ZENBIO | Cat No: 511203 |
| Anti-mouse IgG, HRP-linked Antibody (WB:1/5000) | ZENBIO | Cat No: 511103 |
| TSG101(WB:1/2000) | Abcam | Ca#ab125011 |
| CD63(WB:1/2000) | Abcam | Ca#ab134045 |
| CD81(WB:1/500) | Abcam | Ca#ab79559 |
| α-SMA(WB:1/5000, IHC:1/3000, IF:1/300) | Proteintech | Cat No. 14395-1-AP |
| Collagen Ⅰ (WB:1/2000, IHC:1/1000) | Proteintech | Cat No. 14695-1-AP |
| Fibronectin (IF:1/200) | Proteintech | Cat No. 15613-1-AP |
| TGF-β1 (WB:1/2500) | Proteintech | Cat No. 21898-1-AP |
| E-catenin (WB:1/2000) | Proteintech | Cat No. 12831-1-AP |
| β-catenin (WB:1/1000) | Proteintech | Cat No. 51067-2-AP |
| Vimentin (WB:1/1000) | Cell Signaling | Ca#46173SF |
| Ki67 (IHC:1/200) | Abcam | Ca#ab15580 |
| HNF-4α (IHC:1/400) | BIOSS | Ca#bs-3828R |
| Caspase 3 (IHC:1/1000) | Cell Signaling | Ca#9662S |
| AKT (WB:1/1000, IF:1/300) | Cell Signaling | Ca#4060S |
| PI3K p85 (WB:1/1000) | Cell Signaling | Ca#4257S |
| PI3Kp110 (WB:1/1000) | Cell Signaling | Ca#4249S |
| P-PI3K p85 (WB:1/1000) | Cell Signaling | Ca#17366S |
| P--PI3KCA (WB:1/1000) | BIOSS | Ca#bs-5570R |
| mTOR (WB:1/1000) | Cell Signaling | Ca#2983S |
| Goat anti-rabbit IgG (H+L) secondary antibody, Alexa Fluor 488 (IF:1/350) | Invitrogen | Cat#A-11008 |
| Goat anti-rabbit IgG (H+L) secondary antibody, Alexa Fluor 594 (IF:1/350) | Invitrogen | Cat#A-11012 |
| Goat anti-mouse IgG (H+L) secondary antibody, Alexa Fluor 594 (IF:1/350) | Invitrogen | Cat#A-11005 |

|  |  |  |
| --- | --- | --- |

Table S3 The top 25 significant KEGG pathway between hADMSCs-Exo group (n=3) and regression group (REG, n=3)

| **PathwayID** | **Pathway** | **DEG_number** | **Pvalue** | **FDR** | **Up_gene** | **Down_gene** |
| --- | --- | --- | --- | --- | --- | --- |
| mmu04512 | ECM-receptor interaction | 9 | 8.2626E-07 | 0.000156164 | - | Itga8,Tnc,Lama4,Col6a1,Col6a2,Lamb1,Thbs2,Col1a1,Col1a2 |
| mmu04974 | Protein digestion and absorption | 7 | 0.00017506 | 0.011870001 | - | Col5a2,Col6a1,Col6a2,ln,Col3a1,Col1a1,Col1a2 |
| mmu04510 | Focal adhesion | 10 | 0.00018841 | 0.011870001 | - | Itga8,Myl9,Lama4,Tnc,Col1a1,Col1a2,Col6a1,Lamb1,Col6a2,Thbs2 |
| mmu05146 | Amoebiasis | 6 | 0.0015111 | 0.05711959 | Itgam | Lama4,Col3a1,Lamb1,Col1a2,Col1a1 |
| mmu04933 | AGE-RAGE signaling pathway in diabetic complications | 6 | 0.0015111 | 0.05711959 | Ager | F3,Col3a1,Mmp2,Col1a2,Col1a1 |
| mmu05410 | Hypertrophic cardiomyopathy (HCM) | 5 | 0.00344713 | 0.102162179 | Prkag3,Tnnc1 | Itga8,Des,Tnnt2 |
| mmu05165 | Human papillomavirus infection | 11 | 0.00452849 | 0.102162179 | Cdkn1a,Notch1 | Col6a1,Lamb1,Itga8,Lama4,Tnc,Col6a2,Thbs2,Col1a1,Col1a2 |
| mmu04610 | Complement and coagulation cascades | 5 | 0.00515561 | 0.102162179 | Itgam,Serpina1e | Plat,F3,C7 |
| mmu05222 | Small cell lung cancer | 5 | 0.00592823 | 0.102162179 | Gadd45g,Cdkn1a,Traf1 | Lamb1,Lama4 |
| mmu04710 | Circadian rhythm | 3 | 0.00609178 | 0.102162179 | Cry1,Prkag3 | Nr1d1 |
| mmu05202 | Transcriptional misregulation in cancer | 7 | 0.00630086 | 0.102162179 | Gadd45g,Itgam,Cdkn1a,Cd40,Traf1 | Plat,Igfbp3 |
| mmu00830 | Retinol metabolism | 5 | 0.00648649 | 0.102162179 | Cyp2c38,Cyp4a12b,Cyp4a12a | Ugt1a5,Cyp2c55 |
| mmu04270 | Vascular smooth muscle contraction | 6 | 0.00751686 | 0.109283509 | Cyp4a12a,Cyp4a12b,Npr2 | Actg2,Myl9,Acta2 |
| mmu04115 | p53 signaling pathway | 4 | 0.01199632 | 0.156746139 | Pmaip1,Gadd45g,Cdkn1a | Igfbp3 |
| mmu05033 | Nicotine addiction | 3 | 0.01353466 | 0.156746139 | Gabrb3 | Gabrp,Gabra3 |
| mmu04151 | PI3K-Akt signaling pathway | 10 | 0.01398125 | 0.156746139 | Cdkn1a | Col6a1,Lamb1,Itga8,Tnc,Lama4,Col6a2,Thbs2,Col1a1,Col1a2 |
| mmu04530 | Tight junction | 6 | 0.01409886 | 0.156746139 | Prkag3,Nedd4l | Myh11,Myl9,Tuba1a,Cldn5 |
| mmu05414 | Dilated cardiomyopathy (DCM) | 4 | 0.02022871 | 0.209278703 | Tnnc1 | Des,Tnnt2,Itga8 |
| mmu03320 | PPAR signaling pathway | 4 | 0.0210386 | 0.209278703 | Cyp4a12a,Plin4,Cyp4a12b | Scd1 |
| mmu04926 | Relaxin signaling pathway | 5 | 0.02232451 | 0.210966601 | - | Col1a2,Col1a1,Col3a1,Acta2,Mmp2 |
| mmu00590 | Arachidonic acid metabolism | 4 | 0.02447578 | 0.22028204 | Cyp4a12a,Cyp4a12b,Cyp2c38 | Cyp2c55 |
| mmu00140 | Steroid hormone biosynthesis | 4 | 0.02631428 | 0.22403733 | Cyp2c38 | Akr1c18,Ugt1a5,Cyp2c55 |
| mmu05032 | Morphine addiction | 4 | 0.0272638 | 0.22403733 | Pde1b,Gabrb3 | Gabra3,Gabrp |
| mmu05144 | Malaria | 3 | 0.02994316 | 0.235802407 | Tlr9,Cd40 | Thbs2 |
| mmu05206 | MicroRNAs in cancer | 5 | 0.03126975 | 0.236399326 | Cdkn1a,Notch1,Cdc25c | Reck,Tnc |

Table S4. Significant metabolites between hADMSCs-Exo treatment group (n=6) and regression group (n=6) in the liver tissue samples.

| No. | Name | RT^a^ (min) | *m/z* | VIP | *P*-value^b^ | Fold change (Exo/REG) | Trend^c^ |
| --- | --- | --- | --- | --- | --- | --- | --- |
| 1 | Oleamide | 771.536 | 282.279 | 1.409 | 0.005 | 82.945 | ↑ |
| 2 | Sphingosine | 771.811 | 300.290 | 1.204 | 0.005 | 70.448 | ↑ |
| 3 | FMN | 396.669 | 437.086 | 1.073 | 0.005 | 65.352 | ↑ |
| 4 | Sphinganine | 760.812 | 302.305 | 1.484 | 0.005 | 10.453 | ↑ |
| 5 | Tridemorph | 789.669 | 298.310 | 1.244 | 0.031 | 9.8949 | ↑ |
| 6 | N-a-Acetylcitrulline | 102.761 | 216.098 | 1.315 | 0.031 | 9.7391 | ↑ |
| 7 | Troxilin B3 | 668.421 | 335.224 | 1.374 | 0.031 | 8.7584 | ↑ |
| 8 | Equol | 787.785 | 242.284 | 1.263 | 0.008 | 6.3518 | ↑ |
| 9 | L-Alanyl-gamma-D-glutamyl-L-lysine | 97.477 | 347.191 | 1.656 | 0.008 | 6.1537 | ↑ |
| 10 | 5-Methyl-2-furancarboxaldehyde | 285.204 | 111.044 | 1.368 | 0.031 | 6.0525 | ↑ |
| 11 | 4,5-Dihydroorotic acid | 193.684 | 158.960 | 1.237 | 0.020 | 0.10861 | ↓ |
| 12 | GMP | 90.832 | 362.050 | 1.554 | 0.008 | 0.07309 | ↓ |
| 13 | Raffinose | 90.453 | 487.163 | 1.647 | 0.005 | 0.034457 | ↓ |
| 14 | Acetylcysteine | 63.670 | 162.998 | 1.425 | 0.003 | 0.00048013 | ↓ |
| 15 | S-(Formylmethyl)glutathione | 467.289 | 350.100 | 1.248 | 0.028 | 0.00014934 | ↓ |
| 16 | Pantothenic acid | 369.973 | 220.118 | 1.407 | 0.028 | 3.12E-05 | ↓ |
| 17 | Choline | 111.279 | 104.107 | 1.299 | 0.010 | 2.89E-05 | ↓ |
| 18 | D-Fructose | 489.214 | 161.046 | 1.587 | 0.010 | 6.64E-06 | ↓ |
| 19 | 2-Hydroxy-2-ethylsuccinic acid | 469.011 | 161.046 | 1.342 | 0.010 | 2.45E-06 | ↓ |
| 20 | Betaine | 721.221 | 116.069 | 1.287 | 0.003 | 1.04E-06 | ↓ |

^a^ Retention time of the metabolites during LC-MS.

^b^ *P* values were calculated by Student’s t-test (threshold<0.05)

^c^ Trend, the up and down arrows represent a relative increase and decrease, respectively, in the level of the metabolite in the hADMSCs-Exo treatment group when compared with the regression group.

Table S5. Significant metabolites between hADMSCs-Exo treatment group (n=6) and liver fibrosis group (n=6) in the liver tissue samples.

| No. | Name | RT^a^ (min) | *m/z* | VIP | *P*-value^b^ | Fold change (Exo/LFG) | Trend^c^ |
| --- | --- | --- | --- | --- | --- | --- | --- |
| 1 | FMN | 396.669 | 437.086 | 1.113 | 0.005 | 59.205 | ↑ |
| 2 | Oleamide | 771.536 | 282.279 | 1.349 | 0.005 | 21.429 | ↑ |
| 3 | Troxilin B3 | 668.421 | 335.224 | 1.486 | 0.013 | 17.766 | ↑ |
| 4 | 3alpha,7alpha-Dihydroxy-12-oxo-5beta-cholanate | 752.978 | 389.269 | 1.575 | 0.005 | 15.603 | ↑ |
| 5 | Sphingosine | 771.811 | 300.290 | 1.125 | 0.020 | 13.357 | ↑ |
| 6 | 5-Methyl-2-furancarboxaldehyde | 285.204 | 111.045 | 1.421 | 0.008 | 12.795 | ↑ |
| 7 | N-a-Acetylcitrulline | 102.761 | 216.098 | 1.387 | 0.020 | 11.918 | ↑ |
| 8 | L-Alanyl-gamma-D-glutamyl-L-lysine | 97.477 | 347.191 | 1.658 | 0.005 | 8.5351 | ↑ |
| 9 | Tridemorph | 789.669 | 298.310 | 1.188 | 0.031 | 7.7516 | ↑ |
| 10 | Baicalein | 673.469 | 270.050 | 1.924 | 0.005 | 7.3761 | ↑ |
| 11 | Allocholic acid | 677.197 | 408.285 | 1.333 | 0.045 | 7.1738 | ↑ |
| 12 | Sphinganine | 760.812 | 302.305 | 1.410 | 0.005 | 7.0491 | ↑ |
| 13 | Betaine | 721.221 | 116.069 | 1.715 | 0.003 | 2.36E-06 | ↓ |
| 14 | 2-Hydroxy-2-ethylsuccinic acid | 469.011 | 161.046 | 1.069 | 0.003 | 6.30E-06 | ↓ |
| 15 | D-Galactose | 272.4625 | 161.045 | 1.672 | 0.003 | 6.31E-06 | ↓ |
| 16 | Choline | 111.279 | 104.107 | 1.787 | 0.003 | 9.63E-06 | ↓ |
| 17 | D-Fructose | 489.214 | 220.118 | 1.543 | 0.003 | 1.47E-05 | ↓ |
| 18 | Pantothenic acid | 369.973 | 220.118 | 1.593 | 0.003 | 4.58E-05 | ↓ |
| 19 | S-(Formylmethyl)glutathione | 467.289 | 350.100 | 1.216 | 0.003 | 0.000345 | ↓ |
| 20 | Acetylcysteine | 63.670 | 162.999 | 1.729 | 0.003 | 0.000621 | ↓ |
| 21 | Oleic acid | 794.9885 | 282.279 | 1.333 | 0.004 | 0.005948 | ↓ |
| 22 | S-[(E)-N-Hydroxy(indol-3-yl)acetimidoyl]-L-glutathione | 104.65 | 350.100 | 1.329 | 0.010 | 0.010966 | ↓ |
| 23 | Stearolic acid | 783.715 | 281.248 | 1.806 | 0.005 | 0.051517 | ↓ |
| 24 | Retinol | 781.083 | 269.227 | 1.652 | 0.005 | 0.06952 | ↓ |

^a^ Retention time of the metabolites during LC-MS. ^b^ *P* values were calculated by Student’s t-test (threshold<0.05). ^c^ Trend, the up and down arrows represent a relative increase and decrease, respectively, in the level of the metabolite in the hADMSCs-Exo treatment group when compared with the liver fibrosis group.
